# Adapting and validating the log quadratic model to derive under-five age- and cause-specific mortality (U5ACSM): a preliminary analysis

**DOI:** 10.1186/s12963-021-00277-w

**Published:** 2022-01-10

**Authors:** Jamie Perin, Yue Chu, Francisco Villavicencio, Austin Schumacher, Tyler McCormick, Michel Guillot, Li Liu

**Affiliations:** 1 Department of International Health, Johns Hopkins University, Baltimore, USA; 2 Department of Biostatistics, University of Washington, Seattle, USA; 3 Departments of Statistics and Sociology, University of Washington, Seattle, USA; 4 Department of Sociology, University of Pennsylvania, Philadelphia, USA; 5 Department of Population, Family, and Reproductive Health, Johns Hopkins University, Baltimore, USA

**Keywords:** Under-five mortality, China, Infant mortality, Neonatal mortality, Injury, Pneumonia

## Abstract

**Background:**

The mortality pattern from birth to age five is known to vary by underlying cause of mortality, which has been documented in multiple instances. Many countries without high functioning vital registration systems could benefit from estimates of age- and cause-specific mortality to inform health programming, however, to date the causes of under-five death have only been described for broad age categories such as for neonates (0–27 days), infants (0–11 months), and children age 12–59 months.

**Methods:**

We adapt the log quadratic model to mortality patterns for children under five to all-cause child mortality and then to age- and cause-specific mortality (U5ACSM). We apply these methods to empirical sample registration system mortality data in China from 1996 to 2015. Based on these empirical data, we simulate probabilities of mortality in the case when the true relationships between age and mortality by cause are known.

**Results:**

We estimate U5ACSM within 0.1–0.7 deaths per 1000 livebirths in hold out strata for life tables constructed from the China sample registration system, representing considerable improvement compared to an error of 1.2 per 1000 livebirths using a standard approach. This improved prediction error for U5ACSM is consistently demonstrated for all-cause as well as pneumonia- and injury-specific mortality. We also consistently identified cause-specific mortality patterns in simulated mortality scenarios.

**Conclusion:**

The log quadratic model is a significant improvement over the standard approach for deriving U5ACSM based on both simulation and empirical results.

**Supplementary Information:**

The online version contains supplementary material available at 10.1186/s12963-021-00277-w.

## Background

In the past decade, estimates for the causes of death among young children have become available for all countries, including those where health system and vital registration functioning is low [1, 2]. These estimates are utilized by governments and international organizations for planning health programs, government and program accountability, and other tracking purposes [3]. As governments and the international community increase their investment in developing and implementing age-targeted and disease-specific childhood interventions and policy [4–9], their effectiveness requires more detailed knowledge of the age patterns of under-five mortality and the primary contributors at each age. The majority of these deaths, however, occur in low and middle-income countries (LMICs) without high quality vital registration [1], creating massive uncertainty about age- and cause-specific child mortality. Many LMICs depend on verbal autopsies to examine cause-specific mortality for children under five, either directly through empirical estimates or indirectly through modeled estimates that employ data from verbal autopsy studies [1]. Verbal autopsy studies, however, being among the recently deceased, do not characterize the population sizes by age and so cannot directly estimate cause-specific mortality for small age groups [10].

Developing effective interventions requires understanding of the age at death and from which causes children are dying [1]. For example, the recently announced World Health Organization (WHO) Phase IV clinical trial of RTS, S/AS01 malaria vaccine [9] predicted that if the trial were successful and implemented globally, 18.0% of the 306,000 malaria deaths among children under-5 years of age, or more than 55,000 deaths could be averted [1, 8]. This prediction is based on estimates of malaria mortality at ages 6, 7.5, 9, and 27 months, i.e., the proposed malaria vaccine schedule [8]. However, such age-specific malaria death estimates are likely derived only from malaria high transmission settings where cohort studies of malaria interventions have been conducted. The external validity of such estimates to settings with low transmission or without malaria is questionable. Similarly, age and cause specific mortality is needed to accurately understand the benefits of maternal immunization and pneumococcal pneumonia vaccine [7]. As another example, the Lives Saved Tool, a widely used instrument for health planning in LMICs predicting under five mortality given the coverage of interventions for child health [3], aims to incorporate cause specific mortality distributions for children under five in six age groups: 0–1, 1–5, 6–11, 12–23, and 24–59 months. With high quality estimates of under-five age- and cause-specific mortality (U5ACSM), this tool could be refined in how it selects interventions for the most impact [11]. To precisely estimate the impacts of age-targeted disease-specific interventions, estimates of U5ACSM need systematic development.

Empirical evidence indicates that under-five causes of deaths are not uniform within broad age groups [12–16]. National under-five cause of death estimates for LMICs are available for two broad age categories: 0–27 days and 1–59 months [17]. The global burden of disease study further disaggregated 1–59 months into 1–11 months and 12–59 months [18]. These methods have not comprehensively estimated U5ACSM. Instead, they often estimate cause distributions for broad age groups in different frameworks [17]. In addition, these estimates do not appear to capture sufficient variation in cause of death by age. For example, an estimated 72% of diarrhea and 81% of pneumonia deaths occur in the first 2 years of life [16], with pneumonia- and diarrhea-specific mortality fractions peaking at 0–11 months, then declining substantially at 12–23 months to stabilize at a very low level at 23–59 months. Malaria [12], measles [15], and injury are other examples of causes with uneven age distributions [13]. A complete mortality profile for a given age group would account for the complex interplay of cause contributors to child mortality, across the spectrum of ages under five in a systematic estimation framework.

Although there have not been comprehensive methods to estimate U5ACSM, there are methods for estimating the age structure among all ages from a given population. These methods have been employed for all-cause mortality for demographic purposes such as predicting demographic trends needed for estimating life expectancy, fertility rates, and disparity in life expectancy [19]. In particular, there are several methods predicting age-specific patterns of all-cause mortality among people of all ages with a matrix decomposition approach [20, 21] or given mortality in a selected age group [22–25]. These methods often use under-five mortality as an index for projecting adult mortality, in part because high quality estimates of under-five mortality are available for most areas and over time [26]. We aimed to adapt this general approach used for all-cause mortality to quantify U5ACSM.

The log quadratic model defined by Wilmoth and colleagues is publicly available and has been validated among high quality all-cause mortality data [24]. In addition, the log quadratic model utilizes estimates of under five mortality in a parsimonious and flexible approach. We adapt the log quadratic model here to determine whether U5ACSM can be estimated.

## Methods

### Adapting the log quadratic model

We adapted the log quadratic model between all-cause mortality for adult mortality and under-five all-cause mortality developed by Wilmoth et al. [24]. Specifically, we employed the following log quadratic model,

1 $$\begin{aligned} \log (_{x}q_{0}) = a_{x} + b_{x} \log (_{5}q_{0}) +c_{x} \log (_{5}q_{0})^2 + v_{x}k , \end{aligned}$$

where $_{x}q_{0}$ is the probability of dying from birth up to age *x*, *x* is a preselected age less than 5 years, and log($_{5}q_{0}$) is the logarithm of the probability of dying between birth and age five. We chose *x* to predict $_{x}q_{0}$ in age groups 0–6, 0–27 days, and 0–5, 0–11, 0–23, and 0–59 months to be consistent with the Lives Saved Tool [16], where the upper age limit represents completed days or months, so that 0–59 months is equivalent to the standard under-five mortality rate. The variability of age-specific mortality at a given $_{5}q_{0}$ is represented by $v_{x}$, estimated from the singular value decomposition of the matrix of residuals from the quadratic equation above. The parameter *k* represents the deviation from the average pattern in a life table at a given $_{5}q_{0}$ and can be tailored to fit $_{x}q_{0}$ for a specific age group *x* or to match the mortality over a given age range. The parameter $v_{x}$ is estimated for a reference set of probabilities, while *k* is selected to best fit estimated mortality in a specific life table.

We used probabilities of dying from birth to age *x* for modeling rather than the probability of dying in each age interval ($_{x}q_{0}$ rather than $_{n}q_{y}$, where *n* is the length of the age interval and $x = y + n$). Probabilities of dying from birth to age *x* have the advantage of being more stable. However, violations are possible where predicted $_{x}q_{0}$ may be less than $_{y}q_{0}$ for $0< y < x$, contrary to the interpretation of $_{x}q_{0}$. In the event that these violations are observed, $_{y}q_{0}$ will be restricted by $_{x}q_{0}$ such that $_{y}q_{0}$ < $_{x}q_{0}$ for $0< y < x$. In practice, this type of violation was only observed when $_{x}q_{0}$ and $_{y}q_{0}$ were very similar, when observed mortality between ages *x* and *y* was zero or close to zero. We also focused on the probabilities $_{x}q_{0}$ for specific age groups rather than the mortality rate from birth up to age *x* ($_{x}m_{0}$) as employed by Wilmoth and colleagues. Even though these probabilities ($_{x}q_{0}$) and rates ($_{x}m_{0}$) are closely related, we chose the probability $_{x}q_{0}$ as we observed a smaller coefficient of variation than $_{x}m_{0}$ for under five mortality in our empirical data, which we expected to yield greater model stability. In addition, probabilities $_{x}q_{0}$ are generally more available than rates $_{x}m_{0}$ [26].

Parameters $a_{x}$, $b_{x}$, $c_{x}$, and $v_{x}$ are estimated using Eq. (1), where $_{5}q_{0}$ and $_{x}q_{0}$ for each age group of interest are available from source data, and *k* is estimated for each life table where $_{5}q_{0}$ is known but $_{x}q_{0}$ for smaller age groups is not. We expanded this model to U5ACSM using

2 $$\begin{aligned} \log (_{x}q_{0,c}) = a_{x,c} + b_{x,c} \log (_{5}q_{0,c}) +c_{x,c} \log (_{5}q_{0,c})^2 + v_{x,c} k_c \end{aligned}$$

for age *x* and cause *c*. Here we focused on children of ages 0–6, 0–27 days, and 0–5, 0–11, 0–23, and 0–59 months based on epidemiological evidence [12, 13, 15, 16]. For each of these age groups, we illustrate the  proposed method through pneumonia-specific and injury-specific mortality. We first fitted the adapted log quadratic model using empirical data, then used simulations to address potential measurement issues in the empirical data.

### Empirical validation

We illustrated our adapted methods using mortality data from the Chinese Maternal and Child Health Surveillance System (MCHSS), a sample registration system for child mortality, from the period 1996 until 2015 [27]. The MCHSS was designed to be representative in each of six strata of China, defined by geography (East, Mid, and West) and urbanicity (urban or rural). This system was expanded in 2009 to cover additional population in the age of falling maternal mortality. The livebirths and under-five deaths monitored by this system over time are shown in Additional file 1. Over 80% of causes of death registered in this system were ascertained by medical certification, and the remainder by verbal autopsy [27]. From these data, we aimed to predict $_{x}q_{0}$ and $_{x}q_{0,c}$ for pneumonia and injury.

We used cross validation to examine model performance, estimating these parameters using five of six total strata over the period 1996–2015, and with the resulting parameter values estimated all-cause $_{x}q_{0}$ in the hold out strata using Eq. (1). We examined the average absolute difference between observed and predicted $_{x}q_{0}$, $| \widehat{_{x}q_{0}} \, - \, _{x}q_{0} |$ as well as the average absolute relative difference in the sixth held out stratum,

$$\begin{aligned} \frac{|\widehat{_{x}q_{0}} \, - \, _{x}q_{0} |}{_{x}q_{0}} \end{aligned}$$

We estimated $_{x}q_{0}$ first for the average age-specific mortality profile at a given $_{5}q_{0}$ (when parameter *k* is zero). We then estimated *k* for each life table to match exactly the all-cause neonatal mortality rate for each year in the hold out strata, for another estimate of $_{x}q_{0}$. We compare these estimated $_{x}q_{0}$ to what is typically done when age specific mortality is not available, assuming a constant mortality rate within 0–27 days and 1–59 months [3]. We have labeled these estimates and their associated results as the standard approach.

For U5ACSM, we used observed $_{5}q_{0,c}$ and $_{x}q_{0,c}$ to estimate $a_{x,c}$, $b_{x,c}$, $c_{x,c}$ , and $v_{x,c}$ for deaths due to pneumonia and injury, repeating the above analyses. We used $_{5}q_{0,c}$ as in Eq. (2) to predict a typical $_{x}q_{0,c}$ for age *x*, and we also used neonatal cause-specific mortality when estimating *k* for predicting all $_{x}q_{0,c}$ in a specific life table. Pneumonia and injury were selected because they are among the most common causes of mortality for children under-five in China across the study time period and with a known variation across age [12, 13, 18]. Following the GATHER guideline for international health statistics, data and software to implement the proposed method are available at https://github.com/jamieperin/U5ACSM [28].

### Simulation validation

We also conducted a simulation study to examine the log quadratic model while minimizing the data quality concern associated with the China mortality surveillance system. We generated $_{5}q_{0,c}$ to resemble observed pneumonia mortality in China strata-years, such that $_{5}q_{0,c}$ was uniformly distributed and ranging from 2 deaths per 1000 live births up to 40 deaths per 1000 live births. We then estimated parameters $a_{x,c}$, $b_{x,c}$, $c_{x,c}$ , and $v_{x,c}$ from life tables over six strata in 1996–2015 for pneumonia-specific mortality in China and the log quadratic relationship in Eq. (2). Age- and cause-specific probabilities were generated with varying degrees of error $e_{x,c}$, such that

3 $$\begin{aligned} \log (_{x}q_{0,c}) = a_{x,c} + b_{x,c} \log (_{5}q_{0,c}) +c_{x,c} \log (_{5}q_{0,c})^2 + v_{x,c} k + e_{x,c} , \end{aligned}$$

where $e_{x,c}$ is a normally distributed error term for each age group as observed in China, or with twice the error as in China. We compared parameter estimates to the known parameter values used in (3). We also used parameter estimates from simulated data to predict for an unobserved life table whose pneumonia-specific under five mortality is known in order to estimate the corresponding pneumonia mortality in fine age groups. This estimate of an unobserved life table is the primary interest of the log quadratic model and not the parameter estimates of $a_{x,c}$, $b_{x,c}$, $c_{x,c}$ , and $v_{x,c}$. We selected three values of *k* to represent settings with low, middle, and high neonatal mortality due to pneumonia. We examined prediction error in estimated $_{x}q_{0,c}$ for these hypothetical life tables across 1000 simulations.

## Results

### Empirical validation results

There were over 65 thousand under-five deaths from the China MCHSS in 120 strata-years during the period 1996 to 2015. These data were evaluated for a standardized set of fifteen causes of child mortality, including pneumonia and injury [27]. We used these 120 life tables to investigate the relationship between $_{5}q_{0}$ and $_{x}q_{0}$ for age groups 0–6, 0–27 days, and 0–5, 0–11, 0–23, and 0–59 months. We observed strong linear relationships between all-cause under-five mortality and age-specific mortality for these six age groups (not shown). Through the cross validation, we were able to predict the age-specific all-cause mortality for holdout strata with an average relative error rate of 18% for the highest mortality age group (0–6 days), and as low as 3% for children 0–23 months (Table 1). When estimating shape parameter *k*, we were able to match $_{x}q_{0}$ exactly for 0–27 days when predicting for a holdout stratum, and error in specific age groups generally decreased as a result, from 18% to 16% for neonates 0–6 days, compared to a 67% error using the standard method. In children 0–5-months-old, we were able to estimate all-cause mortality within 3%, compared to 17% in the standard method.

**Table 1:** Relative cross validation error for single hold-out strata in all-cause, pneumonia- and injury-specific mortality by age in China, 1996–2015

Age	Relative error in $_{x}q_{0}$ per 1000 livebirths			_x_*q*_0_ (range)
	Log quadratic with $k =0$ (%)	Log quadratic with estimated *k* (%)	Standard$^{\ddagger }$ (%)	
All cause$^{\dagger }$				
0–6 days ($_{6d}q_{0}$)	18	16	67	1.3–29.7
0–27 days ($_{27d}q_{0}$)	15	0	0	1.9–38.0
0–5 months ($_{5mo}q_{0}$)	7	3	17	2.6–55.3
0–11 months ($_{11mo}q_{0}$)	5	4	18	2.9–62.4
0–23 months ($_{23mo}q_{0}$)	3	3	16	3.2–68.4
0–59 months ($_{59mo}q_{0}$)	0	0	0	3.6–76.7
Pneumonia-specific*				
0–6 days ($_{6d}q_{0,c}$)	78	64	51	0.0–3.5
0–27 days ($_{27d}q_{0,c}$)	47	0	0	0.0–6.9
0–5 months ($_{5mo}q_{0,c}$)	14	11	36	0.1–16.0
0–11 months ($_{11mo}q_{0,c}$)	9	8	37	0.1–20.0
0–23 months ($_{23mo}q_{0,c}$)	5	5	30	0.2–22.9
0–59 months ($_{59mo}q_{0,c}$)	0	0	0	0.2–24.0
Injury-specific**				
0–6 days ($_{6d}q_{0,c}$)	68	62	59	0.0–4.4
0–27 days ($_{27d}q_{0,c}$)	68	0	0	0.0–4.7
0–5 months ($_{5mo}q_{0,c}$)	43	25	31	0.0–6.1
0–11 months ($_{11mo}q_{0,c}$)	32	23	23	0.0–6.5
0–23 months ($_{23mo}q_{0,c}$)	16	17	18	0.1–7.4
0–59 months ($_{59mo}q_{0,c}$)	0	0	0	0.3–11.9

Error shown as average percent difference between estimated and observed $_{x}q_{0}$ and $_{x}q_{0,c}$

${\ddagger }$Based on constant mortality daily/monthly rate across age within 0–27 days and 1–59 months

$\dagger$ $k$ matched to all-cause neonatal mortality

*k matched to pneumonia-specific neonatal mortality

**k matched to injury-specific neonatal mortality

**Fig. 1:**
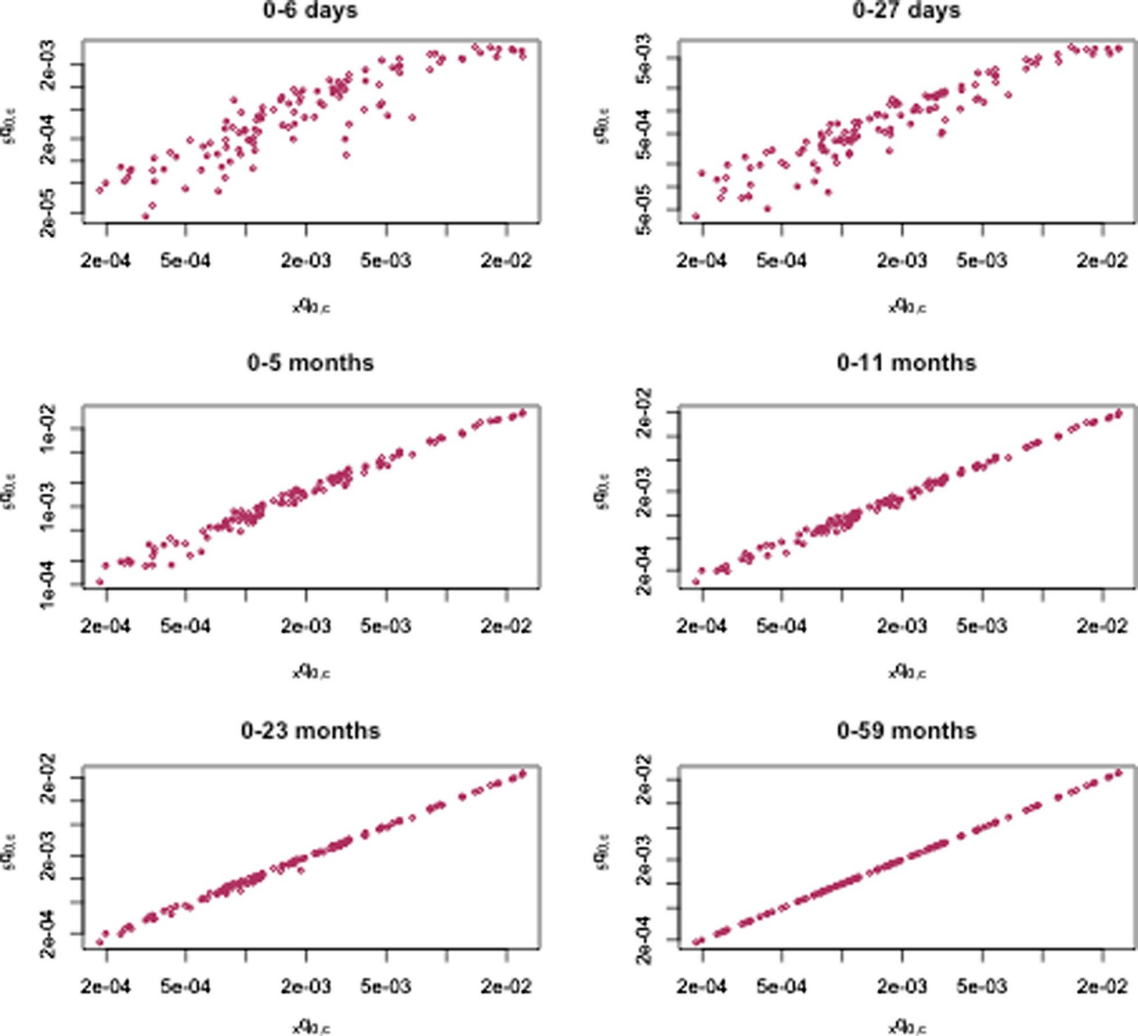
Pneumonia mortality in China by age group. Observed $_{x}q_{0,c}$ and $_{5}q_{0,c}$ for age-specific mortality due to pneumonia in 120 strata years and in six age groups in China, 1996–2015

We repeated these analyses for pneumonia-specific mortality in China using over 10 thousand deaths due to pneumonia. Age- and pneumonia-specific mortality are shown against $_{5}q_{0,c}$ in Fig. 1, where approximate log-linearity is observed, although with apparently more variability than all-cause mortality by age, as expected.

We were able to predict the age- and pneumonia-specific mortality for holdout stratum with an average relative error of 78% for the highest mortality age group (0–6 days), and as low as 5% for children 12–23 months (Table 1). When estimating shape parameter *k* to match pneumonia-specific neonatal mortality, we were able to match pneumonia-specific neonatal mortality exactly for holdout stratum with error decreasing or generally similar for other ages when accounting for this shape, falling from 14 to 11% on average for children age 1–5-months-old, compared to a 36% error with the standard method. For children 6–11-months-old, we were able to estimate pneumonia-specific mortality within 8%, compared to 37% using the standard method.

**Fig. 2:**
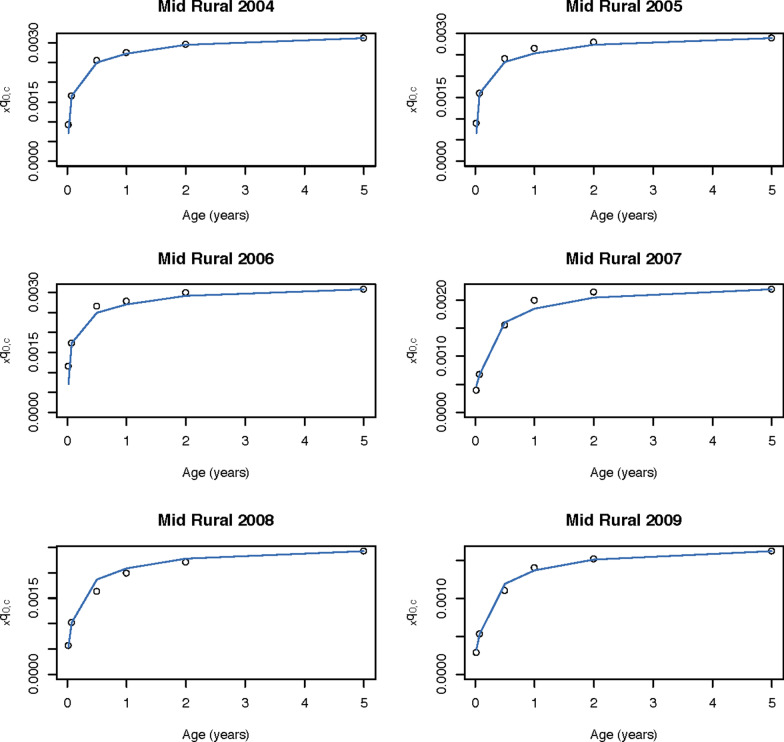
Pneumonia mortaity in China in six strata-years. Observed and predicted $_{x}q_{0,c}$ for age-specific mortality due to pneumonia in the mid rural strata years in China, 2004–2009, estimating shape (*k*) to match pneumonia-specific neonatal mortality exactly

**Fig. 3:**
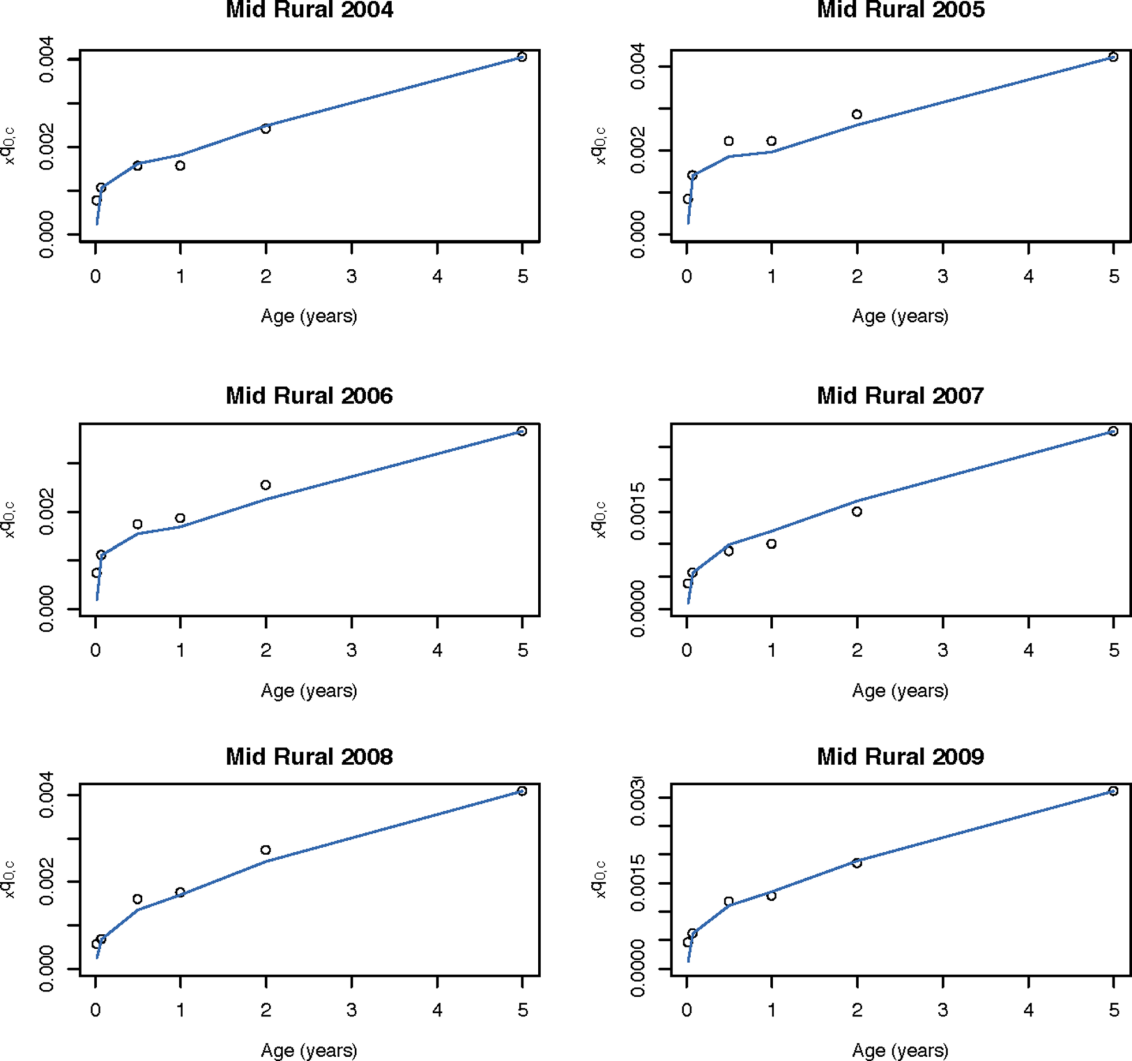
Injury mortality in China in six strata-years. Observed and predicted $_{x}q_{0,c}$ for age-specific mortality due to injury in the mid rural strata years in China, 2004–2009, estimating shape (*k*) to match injury-specific neonatal mortality exactly

We also predicted the age- and injury-specific mortality for holdout stratum with an average relative error of 68% for the highest mortality age group (0–6 days), and as low as 16% per 1000 live births for children 0–23 months (Table 1). When estimating shape parameter *k*, we matched injury-specific neonatal mortality exactly for holdout stratum. Cross validation error for other ages generally decreased when accounting for this shape, falling from 43 to 25% on average for those age 0–5 months, compared to 31% using the standard method. Observed and predicted cause-specific mortality is shown in the mid-rural strata, a moderate mortality area, both observed and predicted, in Figs. 2 and 3 for pneumonia and injury, respectively. Specifically, U5ACSM was predicted for an average age and mortality profile ($k=0$), as well as for each specific life table while estimating shape parameter *k* using neonatal mortality. Predicted U5ACSM is shown for all strata-years for pneumonia in Additional file 2 and injury in Additional file 3.

In addition to relative cross validation error, we also examined absolute error in estimated all-cause, pneumonia- and injury-specific mortality for the log quadratic model compared to the standard method, in Table 2. For all-cause mortality, the standard method always had larger absolute than the log quadratic modeled estimated, in most cases even compared to the log quadratic model assuming a shape (*k*) of 0, or before incorporating neonatal mortality. For injury- and especially pneumonia-specific U5ACSM, cross validation error was always greater using the standard method compared to log quadratic method while estimating shape *k*, with the exception of mortality for the age group 0–6 days. In addition to estimating U5ACSM with the log quadratic model, we have also estimated U5ACSM with a similar model except assuming a log linear association between U5ACSM and $_{5}q_{0}$. These estimates have generally lower cross validation error than the standard approach, however, a few age groups have larger cross validation error compared to the log quadratic model (Additional file 4).

**Table 2:** Average cross validation error for single hold-out strata in all-cause, pneumonia- and injury-specific mortality by age in China, 1996–2015

Age	Absolute error in $_{x}q_{0}$ per 1000 livebirths			_x_*q*_0_ (range)
	Log quadratic with *k* =0	Log quadratic with estimated *k*	Standard$^{\ddagger }$	
All cause$^{\dagger }$				
0–6 days ($_{6d}q_{0}$)	1.92	1.72	6.47	1.3–29.7
0–27 days ($_{27d}q_{0}$)	1.99	0.00	0.00	1.9–38.0
0–5 months ($_{5mo}q_{0}$)	1.03	0.68	2.68	2.6–55.3
0–11 months ($_{11mo}q_{0}$)	0.75	0.81	3.21	2.9–62.4
0–23 months ($_{23mo}q_{0}$)	0.43	0.60	3.00	3.2–68.4
0–59 months ($_{59mo}q_{0}$)	0.00	0.00	0.00	3.6–76.7
Pneumonia-specific*				
0–6 days ($_{6d}q_{0,c}$)	0.65	0.50	0.43	0.0–3.5
0–27 days ($_{27d}q_{0,c}$)	0.71	0.00	0.00	0.0–6.9
0–5 months ($_{5mo}q_{0,c}$)	0.37	0.21	1.04	0.1–16.0
0–11 months ($_{11mo}q_{0,c}$)	0.26	0.20	1.21	0.1–20.0
0–23 months ($_{23mo}q_{0,c}$)	0.11	0.10	1.08	0.2–22.9
0–59 months ($_{59mo}q_{0,c}$)	0.00	0.00	0.00	0.2–24.0
Injury-specific**				
0–6 days ($_{6d}q_{0,c}$)	0.23	0.16	0.29	0.0–4.4
0–27 days ($_{27d}q_{0,c}$)	0.34	0.00	0.00	0.0–4.7
0–5 months ($_{5mo}q_{0,c}$)	0.27	0.14	0.23	0.0–6.1
0–11 months ($_{11mo}q_{0,c}$)	0.19	0.16	0.17	0.0–6.5
0–23 months ($_{23mo}q_{0,c}$)	0.14	0.14	0.22	0.1–7.4
0–59 months ($_{59mo}q_{0,c}$)	0.00	0.00	0.00	0.3–11.9

Error shown as average absolute difference between estimated and observed $_{x}q_{0}$ and $_{x}q_{0,c}$

${\ddagger }$Based on constant mortality daily/monthly rate across age within 0–27 days and 1–59 months

$\dagger$ $k$ matched to all-cause neonatal mortality

*k matched to pneumonia-specific neonatal mortality

**k matched to injury-specific neonatal mortality

### Simulation validation results

**Fig. 4:**
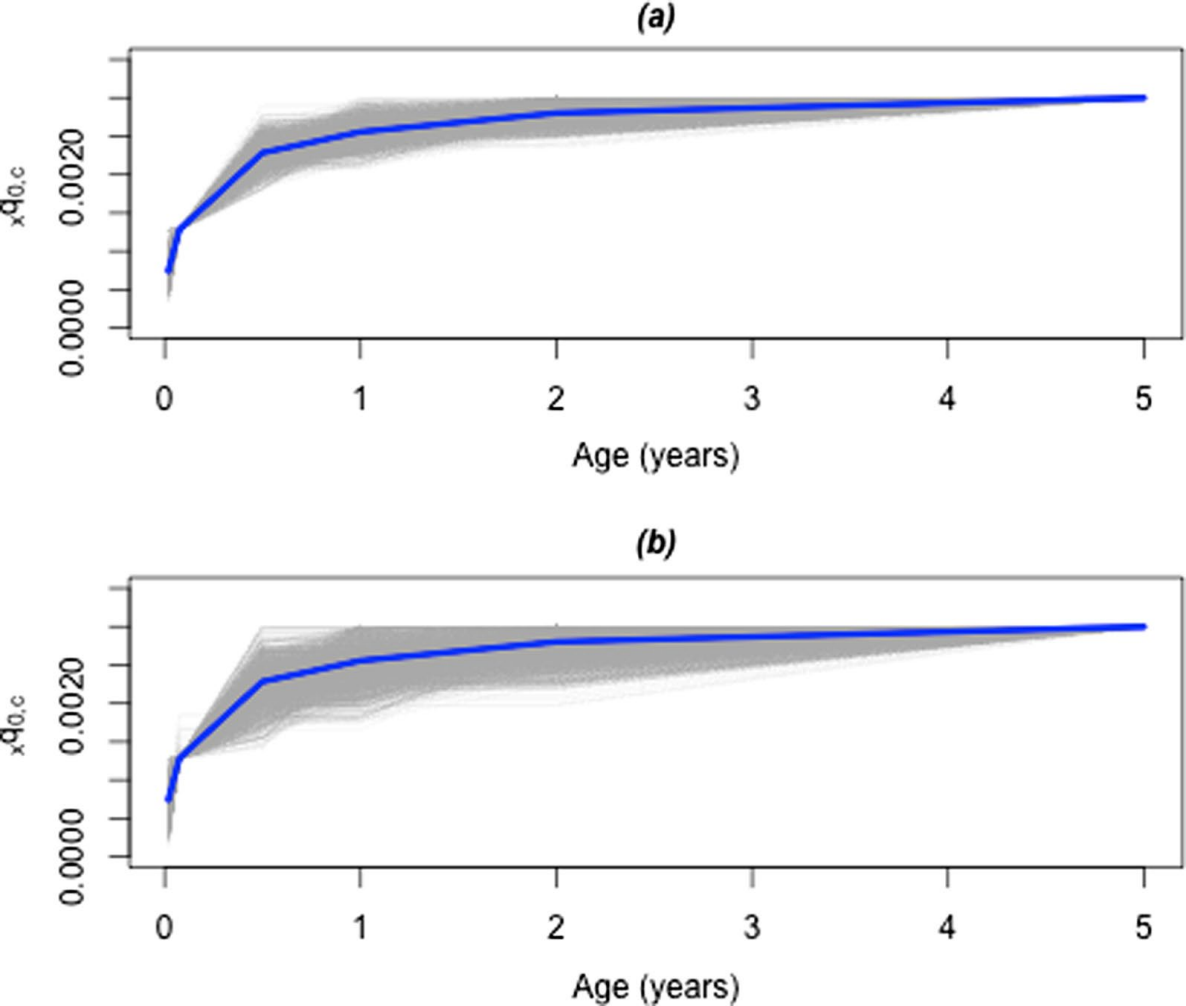
Simulated mortality based on pneumonia mortality in China. Cause-specific mortality predicted from simulated data, where the true age and mortality profile for a high mortality area are shown in blue, and the age and cause specific mortality generated by (3) and estimated by (2), including shape parameter *k*, in 1000 simulations shown in grey, for error in mortality by age comparable to that observed in China (**a**) and for approximately twice the error by age as observed in China (**b**)

Across 1000 simulations, we observed consistent estimation of the parameters $a_{x,c}$, $b_{x,c}$, $c_{x,c}$ , and $v_{x,c}$, with absolute error close to zero and a minimal deviation, especially for $a_{x,c}$, $b_{x,c}$, and $c_{x,c}$ (not shown). However, the primary interest is in the quality of the predictions in “out of sample” life tables for the hypothetical setting. We predicted age-specific mortality for low, moderate, and high mortality, with $_{5}q_{0,c}$ of 1, 2, and 3 deaths per 1000 livebirths, respectively, taken from the 25th, 50th, and 75th percentiles of the distribution of $_{5}q_{0,c}$ for pneumonia in the China strata-years. As expected, the relative prediction error in the low mortality setting was very low (not shown). Results are shown in Fig. 4 for the high mortality scenario, for error approximately equal to that in the China MCHSS (Fig. 4a), and for error approximately twice that seen in China (Fig. 4b). Although the general shape of pneumonia-specific mortality is replicated, when the probabilities $_{x}q_{0,c}$ are observed with an error similar to that in China, we see that predicted mortality in older children is closer to the truth than for younger children, as shown by the grey lines being more closely clustered around the blue line. When measurement error in observed $_{x}q_{0,c}$ is roughly twice that of China, prediction accuracy decreases for all ages, including neonatal age groups, shown in Fig. 4 for a high mortality scenario, having under five mortality due to pneumonia at 3 per 1000 livebirths. Simulated results for low and moderate mortality settings were similar or with lower cross validation error (not shown).

## Discussion

We adapted a log quadratic model that has been used for predicting adult mortality to under-five age-specific all-cause and cause-specific mortality which significantly outperformed the current standard. To our knowledge, this is the first attempt to estimate all-cause and cause-specific mortality at this age resolution. We applied this adapted model to life tables available for six strata in China in 1996–2015 from mortality surveillance with medium quality. We also simulated life tables to make predictions in an environment where the “truth” and the measurement error are known to better understand estimates’ behavior.

We were able to advance upon this goal to estimate age-specific patterns in all-cause and cause-specific mortality with some success using the MCHSS data from China. We predicted pneumonia- and injury-specific mortality in six age groups for children under five with an error rate ranging from 0.1 to 0.7 deaths per 1000 live births, compared to error as high as 1.2 in the standard approach, which ignored the variation in cause-specific mortality across age. The standard approach was slightly better among the youngest neonates for both pneumonia- and injury-specific mortality, however, in older children the proposed method had as little as one tenth the error of the standard approach in pneumonia-specific mortality (0.10 vs. 1.08 per 1000 livebirths). Even in injury-specific mortality, which appears more consistent across age than pneumonia, the proposed method outperformed the standard approach in three age groups by as much as 40% (0.14 vs. 0.23). In all-cause mortality, the proposed method outperformed the standard approach in absolute and relative error by a fair margin in every age group considered. We demonstrated similar prediction error as that in the empirical MCHSS data for the proposed method in life tables simulated to resemble specific mortality scenarios across several different overall levels of pneumonia mortality.

We expected multifaceted challenges in achieving our aim. The age of children is often rounded in reporting, a phenomenon known as age heaping, which could impact consistency and uncertainty in any estimation framework. We also utilized causes of death determined by verbal autopsy, which is generally subject to error [29]. A flexible approach could accommodate these challenges but would also need to maximize transparency and interpretability for sharing with other researchers and those using U5ACSM estimates [28]. We did not expect to address all these challenges (age heaping, error in cause of death assignment, transparency) in a first attempt but instead to identify a method that could be adapted progressively.

Our study was limited by the quality of the MCHSS information from China, which is not equal to that of many well-functioning vital registration systems. Despite this limitation, we were able to identify consistent patterns across age for pneumonia and injury-specific mortality. Higher quality vital registration mortality information, such as that in high income countries, could provide insight into heterogeneity in patterns of mortality across age with less data quality concerns. This analysis for mortality in medium quality mortality data, however, provides greater insight into issues of data quality that are expected in areas with incomplete vital registration, where the proposed methods would be most useful. In addition to these limitations, although we considered multiple causes of mortality, we did not constrain our analysis by all-cause mortality. Although post-hoc methods could be applied to multiple cause estimates for reconciliation with all-cause estimates, such as a pro-rata scaling across causes, more research is needed to determine the best methods to make U5ACSM estimates consistent with all-cause mortality.

We were also limited by our empirical estimates of under-five mortality, which were not based on the true under-five population size, but rather on the number of live births in each year due to the limitations of the MCHSS. As a consequence, mortality for older children may not be estimated as well as that for younger children. However, we do not expect this limitation to impact the proposed method more than the standard approach. We have conducted a sensitivity analysis for reconstructed birth cohorts in a limited set of years and repeated our cross validation analysis for all cause mortality (Additional file 5), with similar results. More research is needed in other settings to determine the behavior and applicability of the proposed methods and their generalizability.

Our proposed method outperformed a standard approach for the MCHSS data and therefore are likely the most appropriate way to predict U5ACSM for these age groups when they are unknown. However, our analysis here did not include all specified causes of under-five mortality, and so prediction error may be higher for unexamined causes, particularly for those with low mortality. We also have not examined prediction error for these methods in a diverse geography or time span where there is more heterogeneity in health programs. We expect some variation in health programs such as differences in the coverage of interventions to be reflected in $_{5}q_{0,c}$, but future research is needed to determine the applicability of the proposed methods in more diverse settings.

It is also possible that there is a more favorable adaptation of Wilmoth’s method that could be used to estimate U5ACSM. In addition to the adaptation proposed here, we examined the relationship between neonatal mortality and U5ACSM, as well as neonatal mortality in conjunction with $_{5}q_{0}$. We also examined all-cause $_{5}q_{0}$ as a predictor of cause-specific $_{x}q_{0,c}$, although we did not observe an improvement in our predicted U5ACSM with these different adaptations.

With the proposed adaptation of Wilmoth’s method, it is possible to estimate U5ACSM for small age groups given an area’s cause-specific under-five mortality and with greater accuracy given that area’s cause-specific neonatal mortality. In areas where estimated cause-specific under five mortality is estimated with lower quality, the predictive ability of the proposed method may also be lower. To develop U5ASCM in these settings, an appropriate reference would need to be established, which may be available from birth histories in household surveys for children under five. Much detail regarding age and timing of mortality is recorded in household surveys, some of which include verbal autopsy for determining causes of mortality, possibly in sufficient detail to contribute to model building for U5ACSM [30]. More research is needed to determine how quality concerns such as age heaping would affect estimates, and whether such data can be used to estimate U5ACSM in LMICs.

We observed high rates of variability among the youngest age groups (0–6d and 7–27d, Fig. 1), as well as high cross validation error both for the proposed approach and using standard methods. This may be due in part to measurement errors in these younger age groups [30, 31]. These could include age heaping at 7d and 27d or misclassification between stillbirth and neonatal death [17], as well as between the causes of death and the underreporting of neonatal mortality. Improving the measurement of all-cause and cause-specific neonatal mortality would help improve the estimation of age-specific all-cause and cause-specific mortality. The methods proposed here also have potential in data quality assessment for empirical age- and cause-specific mortality at the population level for verbal autopsy studies, where data for individuals and site-specific validation are often not available.

## Conclusions

Future methods of cause of death estimation for small age groups in areas with insufficient vital registration will likely benefit from ongoing research. Methods presented here have the potential to extend to other causes of child mortality with different patterns across age. Mortality surveillance in areas with historically low quality vital registration is increasing with studies such as the Child Health and Mortality Prevention Surveillance (CHAMPS) [32] and the Comprehensive Mortality Surveillance for Action (COMSA) [33]. These studies incorporate new technology in the minimally invasive tissue sampling (MITS) that may increase the usefulness of verbal autopsies, and so provide additional high quality mortality information for methods such as ours. In particular, cause ascertainment could be improved thus reducing error in causes determined by verbal autopsy.

Most of the estimated 5.9 million deaths for children under five in 2015 occurred in areas where information is scarce [1]. Given that most child deaths are unrecorded and their primary cause unknown, improvements for vital registration systems in vulnerable areas is a top priority. However, the time and investment necessary to record and ascertain the occurrence and cause of death in young children in these areas is substantial. While vital and health systems are working to register all under five deaths, we have an opportunity to leverage information already available from areas with high and medium quality registration. If age and cause patterns were better understood, they have the potential to refine current tools and guide further reduction of child mortality in the era of the Sustainable Development Goals [34].

## Supplementary Information

**Additional file 1.** Births and Deaths in the China MCHSS.

**Additional file 2.** Estimated U5ACSM for pneumonia-specific mortality in the China MCHSS.

**Additional file 3.** Estimated U5ACSM for injury-specific mortality in the China MCHSS.

**Additional file 4.** Estimated U5ACSM using log linear model.

**Additional file 5.** Sensitivity to birth cohorts in cross validation for analysis of all cause mortality.

## Data Availability

The data and software used in these estimates are available at https://github.com/jamieperin/U5ACSM.

## Abbreviations

U5ACSM: Under-five age-and cause-specific mortality
LMICs: Low and middle-income countries
WHO: World Health Organization
MCHSS: Chinese Maternal and Child Health Surveillance System
DHS: Demographic and health survey
CHAMPS: Child health and mortality prevention surveillance
COMSA: Comprehensive mortality surveillance for action
MITS: Minimally invasive tissue sampling
