# Correction to: Adapting and validating the log quadratic model to derive under-five age and cause-specific mortality (U5ACSM): a preliminary analysis

**DOI:** 10.1186/s12963-022-00292-5

**Published:** 2022-06-28

**Authors:** Jamie Perin, Yue Chu, Francisco Villavicencio, Austin Schumacher, Tyler McCormick, Michel Guillot, Li Liu

**Affiliations:** 1 Department of International Health, Johns Hopkins University, Baltimore, USA; 2 Department of Biostatistics, University of Washington, Seattle, USA; 3 Departments of Statistics and Sociology, University of Washington, Seattle, USA; 4 Department of Sociology, University of Pennsylvania, Philadelphia, USA; 5 Department of Population, Family, and Reproductive Health, Johns Hopkins University, Baltimore, USA

## Correction to: Population Health Metrics (2022) 20:3 10.1186/s12963-021-00277-w

Following publication of the original article [1], it was reported that Francisco Villavicencio’s name was misspelled as ‘Villaviciencio’.

The correct name is given in the authorship panel of this correction, and the original article [1] has been updated.
