# Supplementary figures and images for: Adapting and validating the log quadratic model to derive under-five age- and cause-specific mortality (U5ACSM): a preliminary analysis
Source: Popul Health Metr. 2022 Jan 10;20:3. doi: 10.1186/s12963-021-00277-w (PMC8744238; doi:10.1186/s12963-021-00277-w)

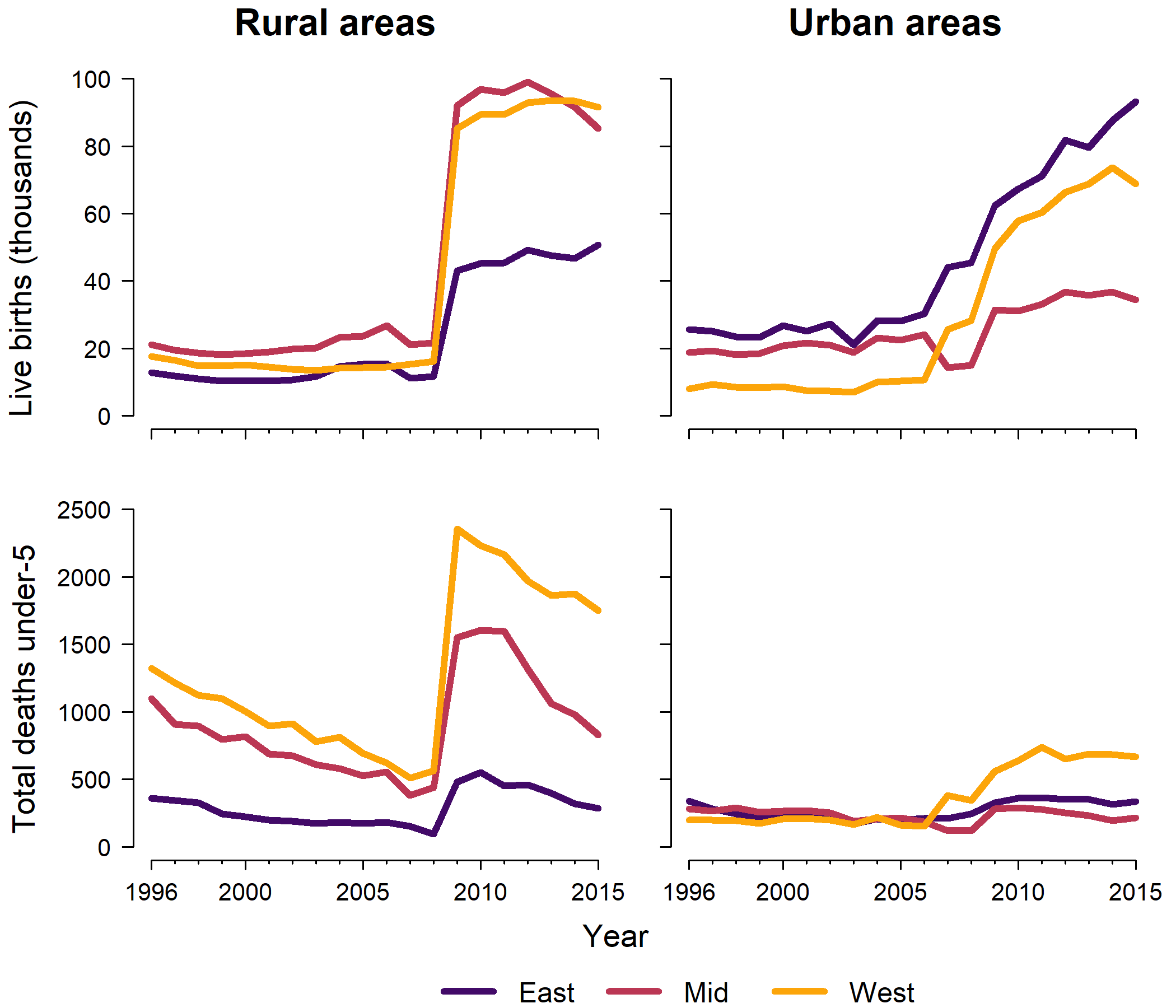

Supplement: Supplementary file 1 — Additional file 1. Births and Deaths in the China MCHSS. [file 12963_2021_277_MOESM1_ESM.png]
