# Supplementary material for: Adapting and validating the log quadratic model to derive under-five age- and cause-specific mortality (U5ACSM): a preliminary analysis
Source: Popul Health Metr. 2022 Jan 10;20:3. doi: 10.1186/s12963-021-00277-w (PMC8744238; doi:10.1186/s12963-021-00277-w)
Supplement: Supplementary file 3 — Additional file 3. Estimated U5ACSM for injury-specific mortality in the China MCHSS. [file 12963_2021_277_MOESM3_ESM.pdf]

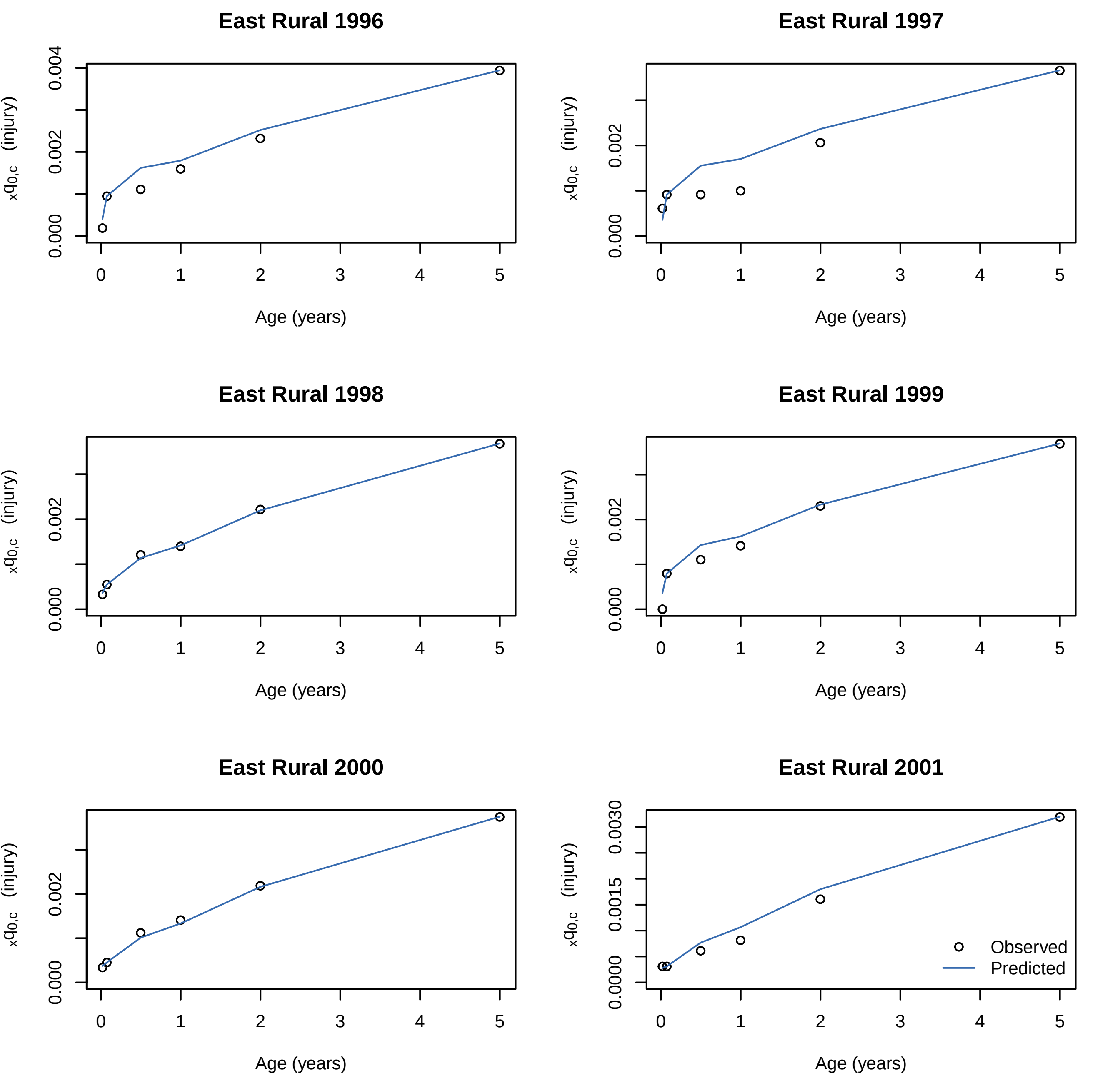
East Rural 1996
East Rural 1997
East Rural 1998
East Rural 1999
East Rural 2000
East Rural 2001
$_{x}q_{0,c}$ (injury)
Age (years)
0.000
0.002
0.004
0.0000
0.0015
0.0030
0 1 2 3 4 5
Observed
Predicted

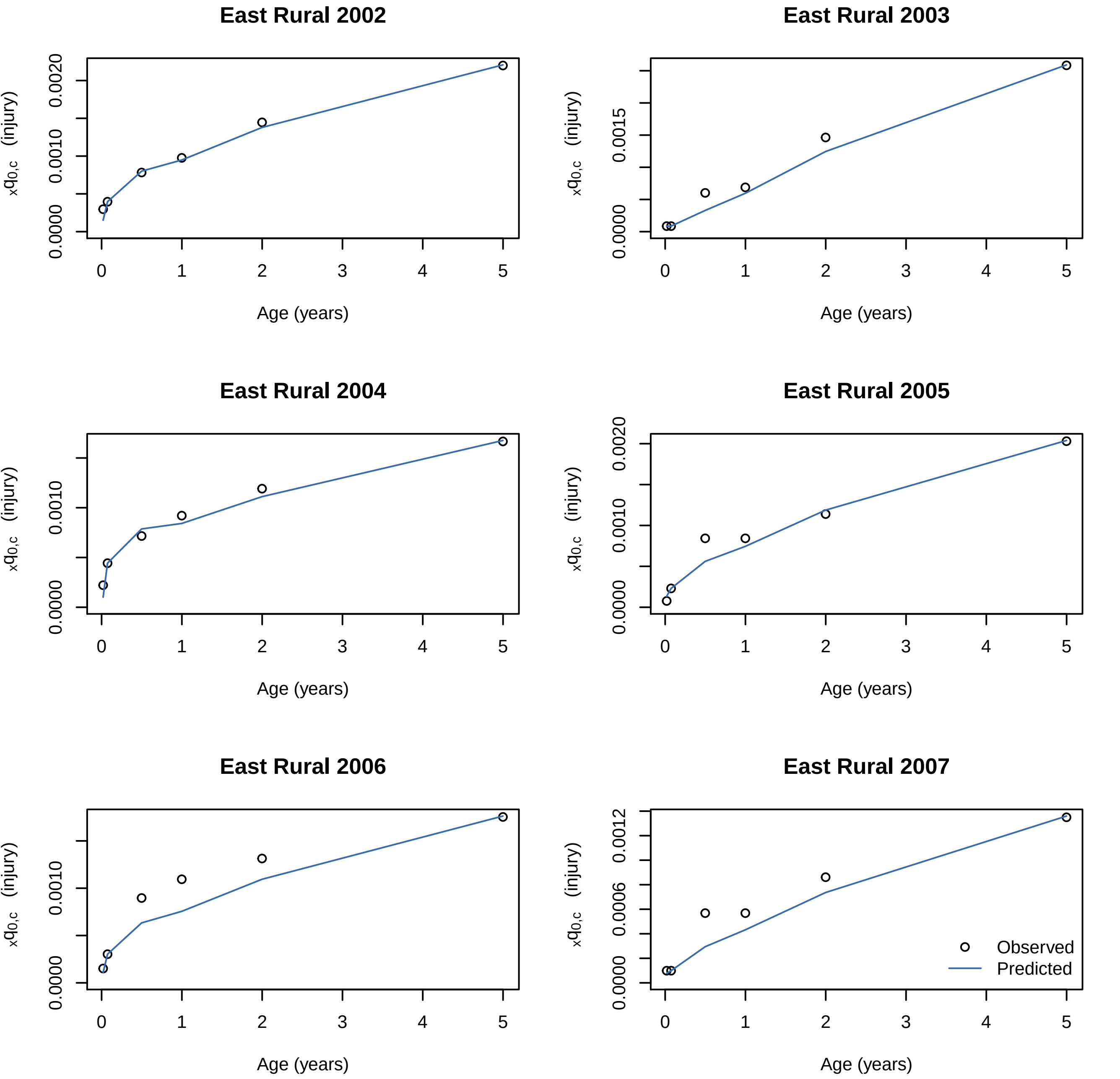
East Rural 2002
0.0000
0.0010
0.0020
$_{x}q_{0,c}$ (injury)
0
1
2
3
4
5
Age (years)
East Rural 2003
0.0000
0.0015
$_{x}q_{0,c}$ (injury)
0
1
2
3
4
5
Age (years)
East Rural 2004
0.0000
0.0010
$_{x}q_{0,c}$ (injury)
0
1
2
3
4
5
Age (years)
East Rural 2005
0.0000
0.0010
0.0020
$_{x}q_{0,c}$ (injury)
0
1
2
3
4
5
Age (years)
East Rural 2006
0.0000
0.0010
$_{x}q_{0,c}$ (injury)
0
1
2
3
4
5
Age (years)
East Rural 2007
0.0000
0.0006
0.0012
$_{x}q_{0,c}$ (injury)
0
1
2
3
4
5
Age (years)
Observed
Predicted

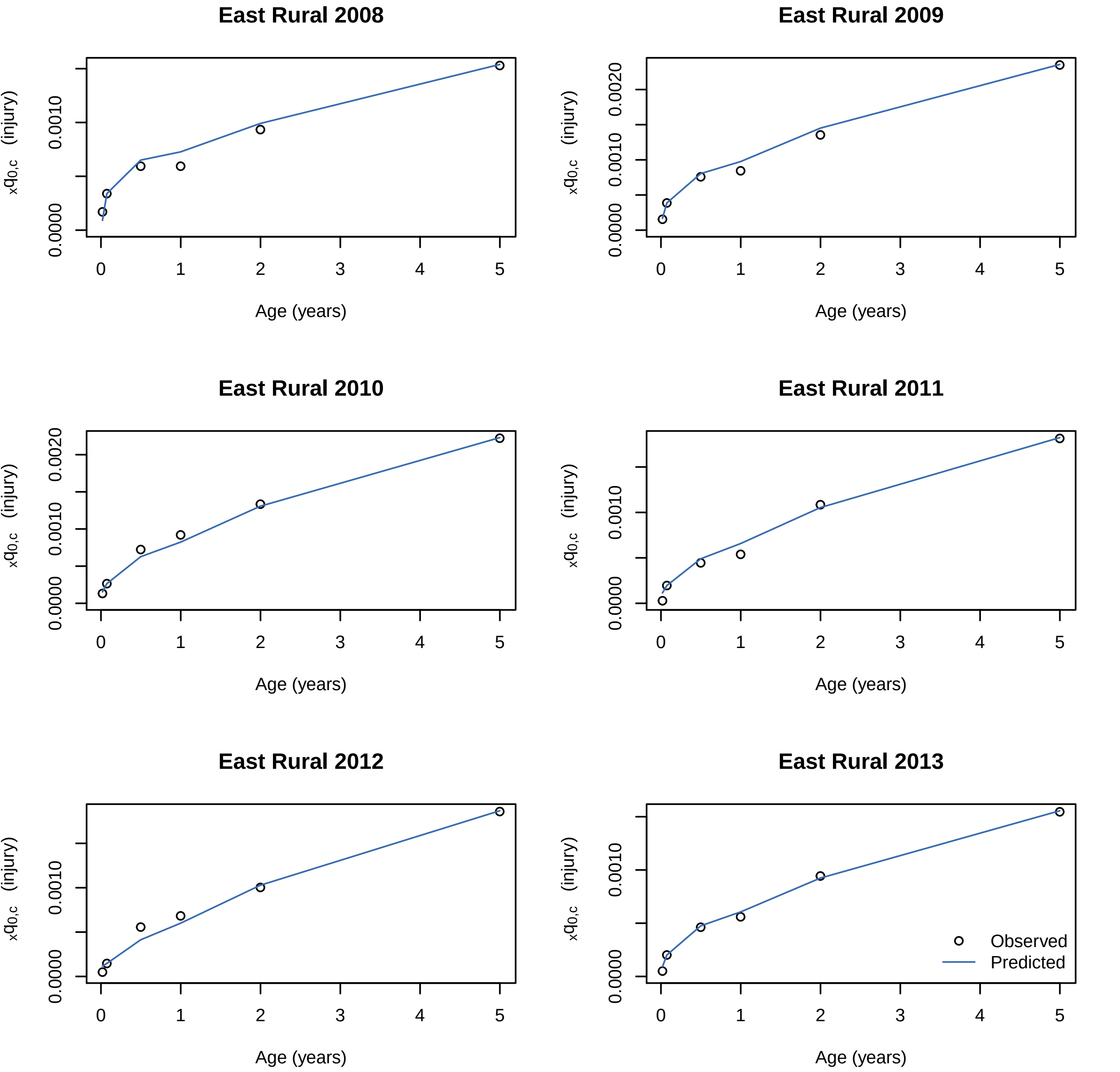
East Rural 2008
East Rural 2009
East Rural 2010
East Rural 2011
East Rural 2012
East Rural 2013
xq0,c (injury)
0.0000
0.0010
0.0020
0 1 2 3 4 5
Age (years)
Observed
Predicted

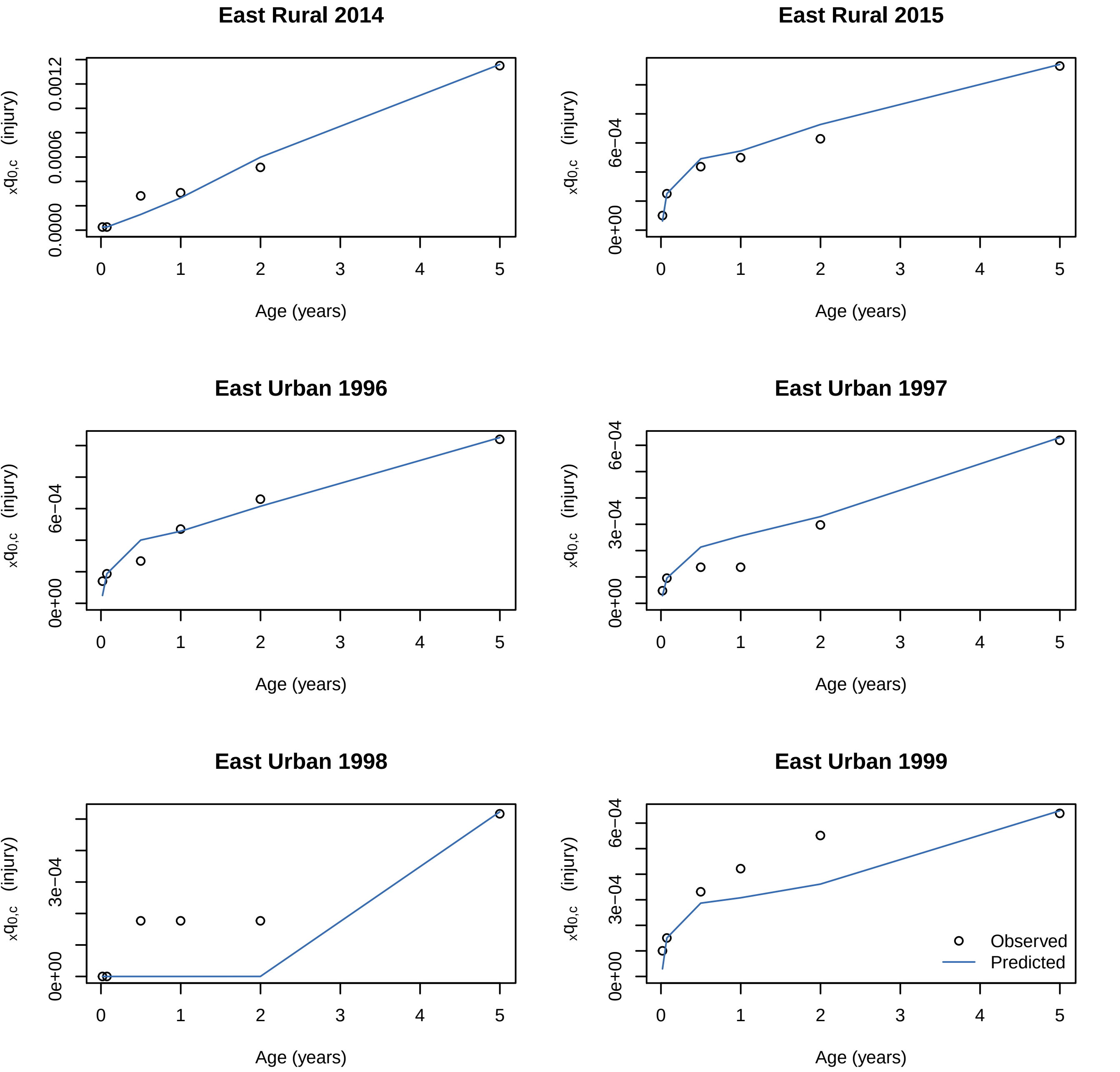
East Rural 2014
East Rural 2015
East Urban 1996
East Urban 1997
East Urban 1998
East Urban 1999
$_xq_{0,c}$ (injury)
Age (years)
0.0000
0.0006
0.0012
0e+00
3e−04
6e−04
0
1
2
3
4
5
Observed
Predicted

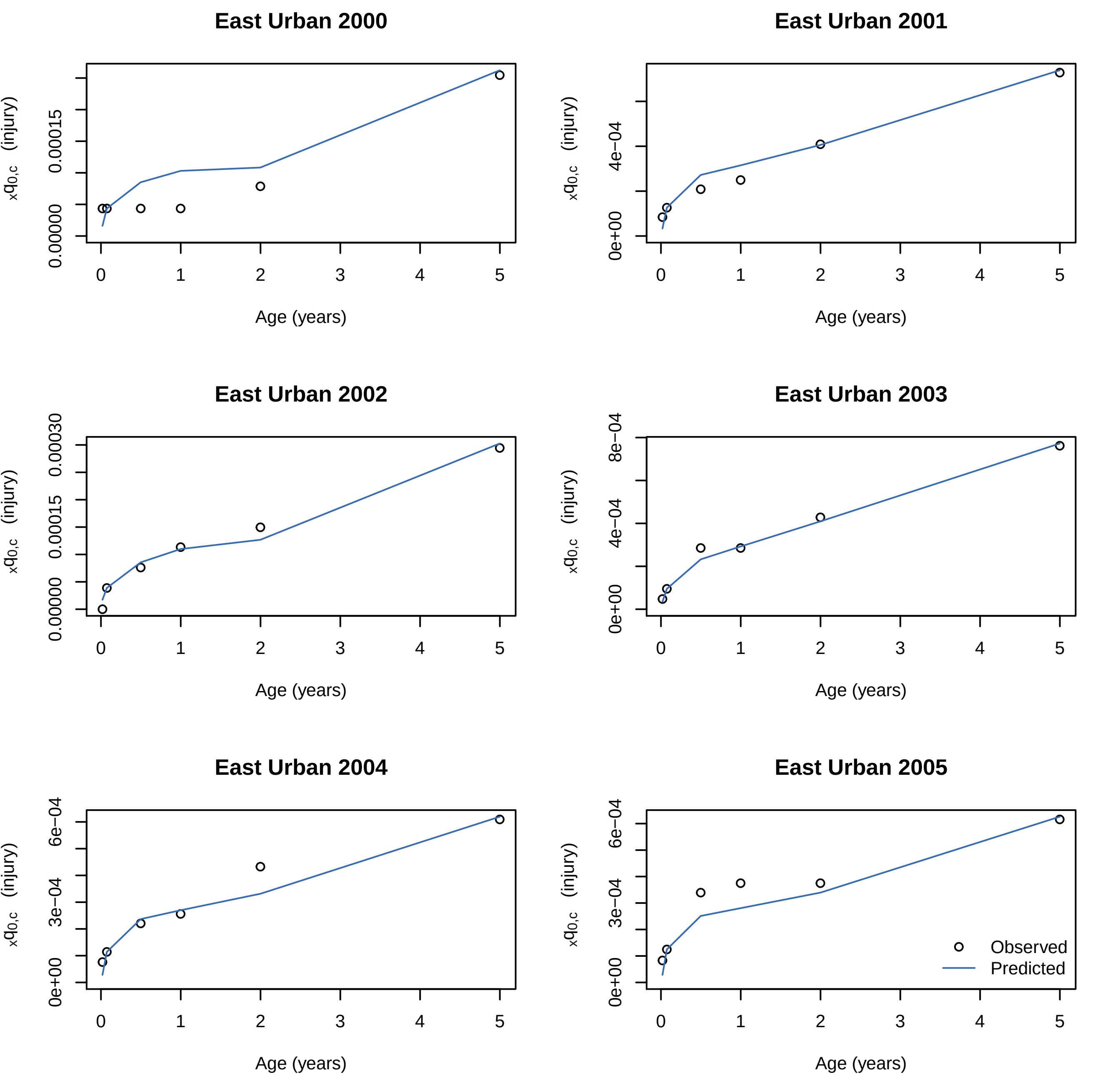
East Urban 2000
East Urban 2001
East Urban 2002
East Urban 2003
East Urban 2004
East Urban 2005
$_xq_{0,c}$ (injury)
Age (years)
0.00000
0.00015
0.00030
0e+00
4e−04
8e−04
3e−04
6e−04
0
1
2
3
4
5
Observed
Predicted

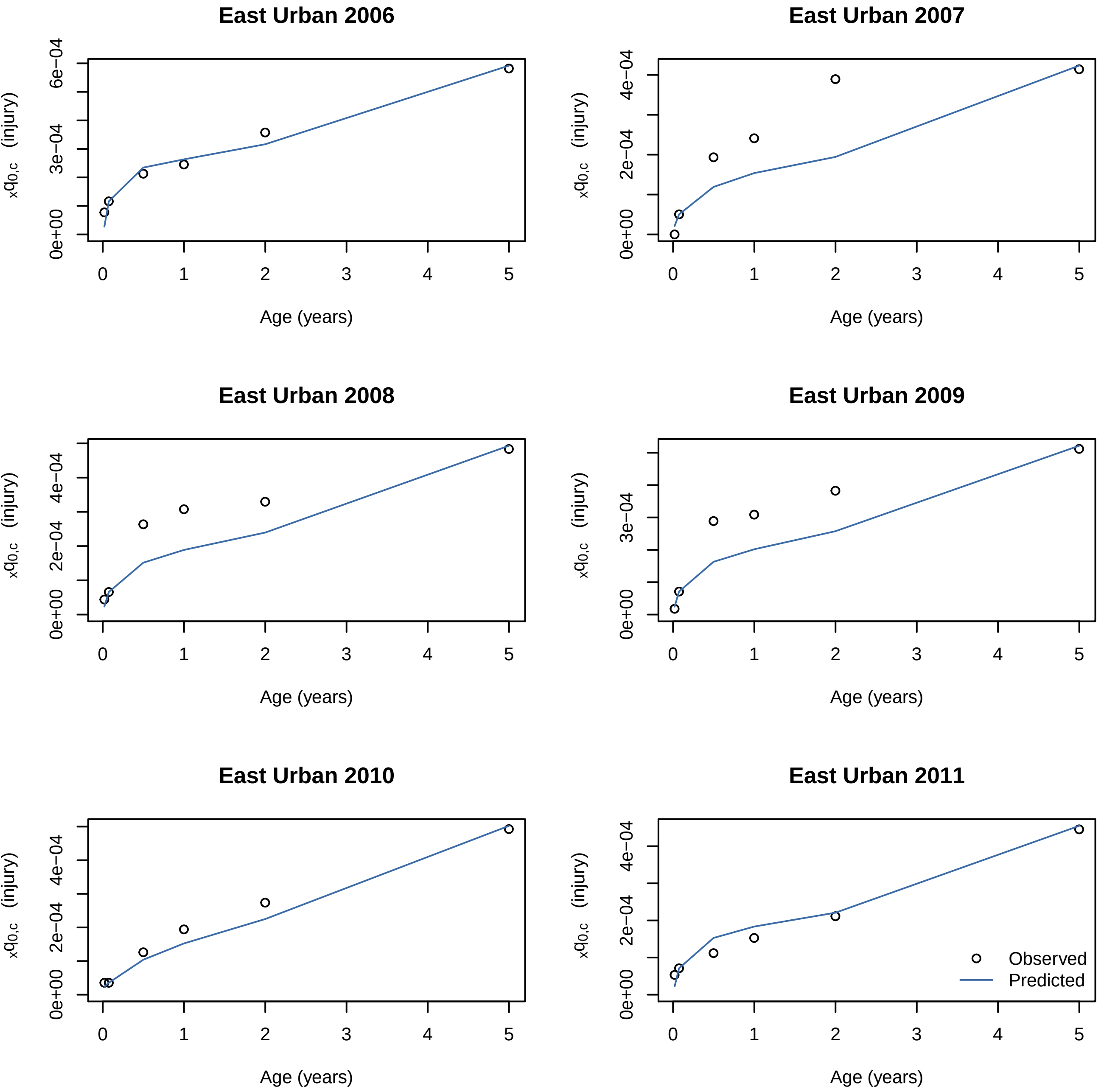
East Urban 2006
East Urban 2007
East Urban 2008
East Urban 2009
East Urban 2010
East Urban 2011
${}_{x}q_{0,c}$ (injury)
Age (years)
0e+00
3e−04
6e−04
2e−04
4e−04
0
1
2
3
4
5
Observed
Predicted

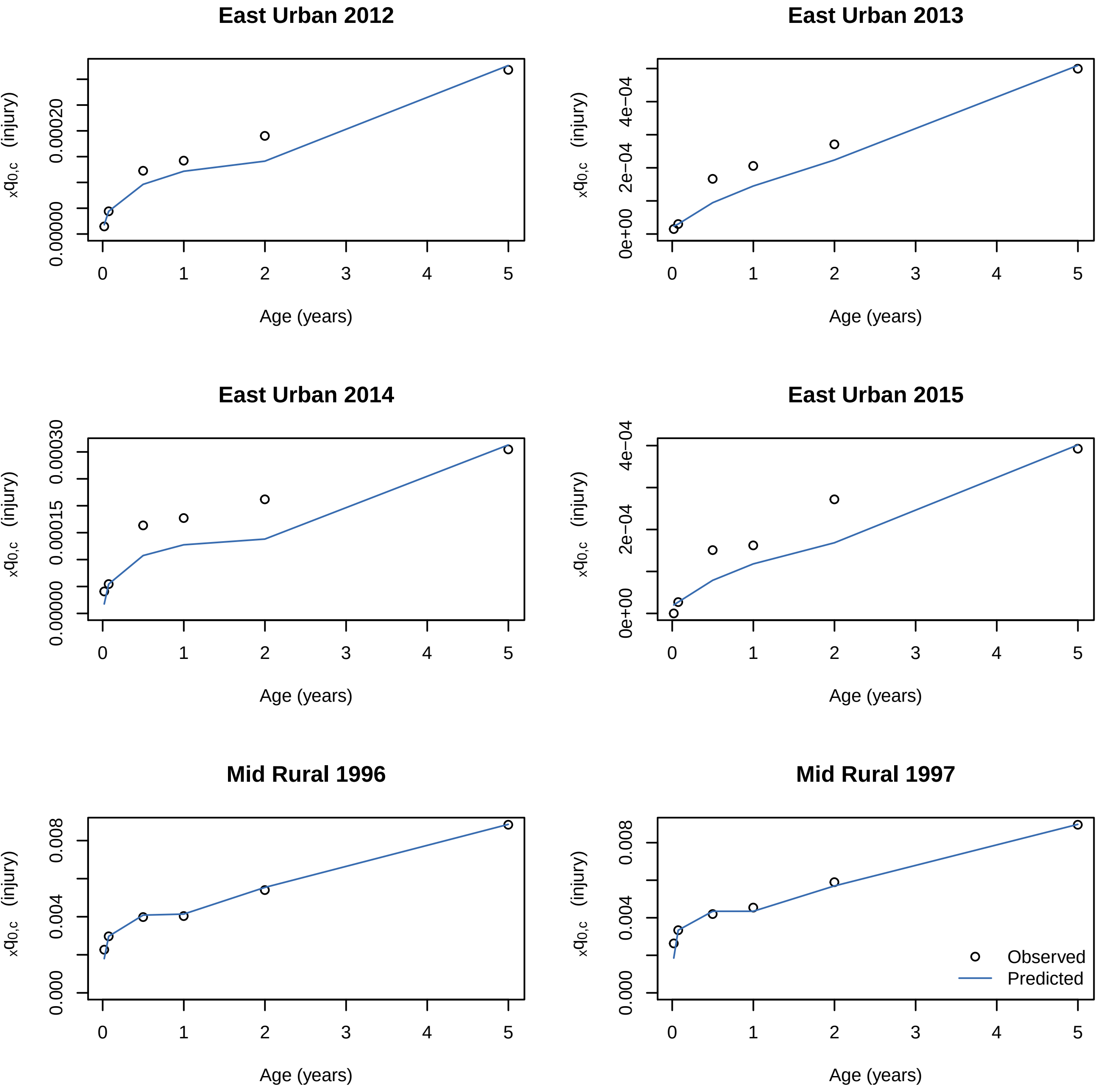
East Urban 2012
East Urban 2013
East Urban 2014
East Urban 2015
Mid Rural 1996
Mid Rural 1997
$_xq_{0,c}$ (injury)
Age (years)
0.00000
0.00020
0.00015
0.00030
0e+00
2e−04
4e−04
0.000
0.004
0.008
0
1
2
3
4
5
Observed
Predicted

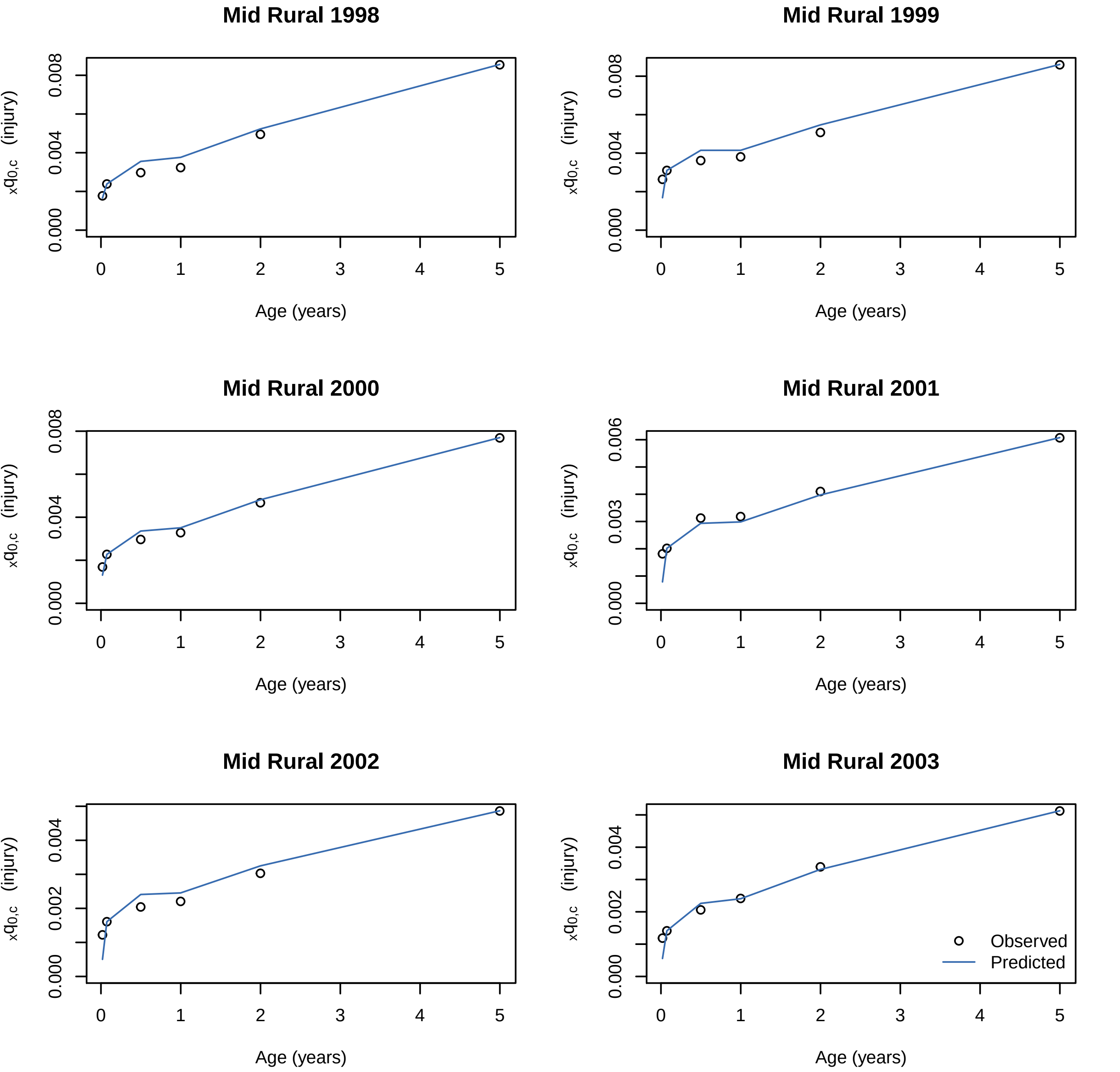

Mid Rural 1998
Mid Rural 1999
Mid Rural 2000
Mid Rural 2001
Mid Rural 2002
Mid Rural 2003
$_xq_{0,c}$ (injury)
Age (years)
0.000
0.002
0.003
0.004
0.006
0.008
0 1 2 3 4 5
Observed
Predicted

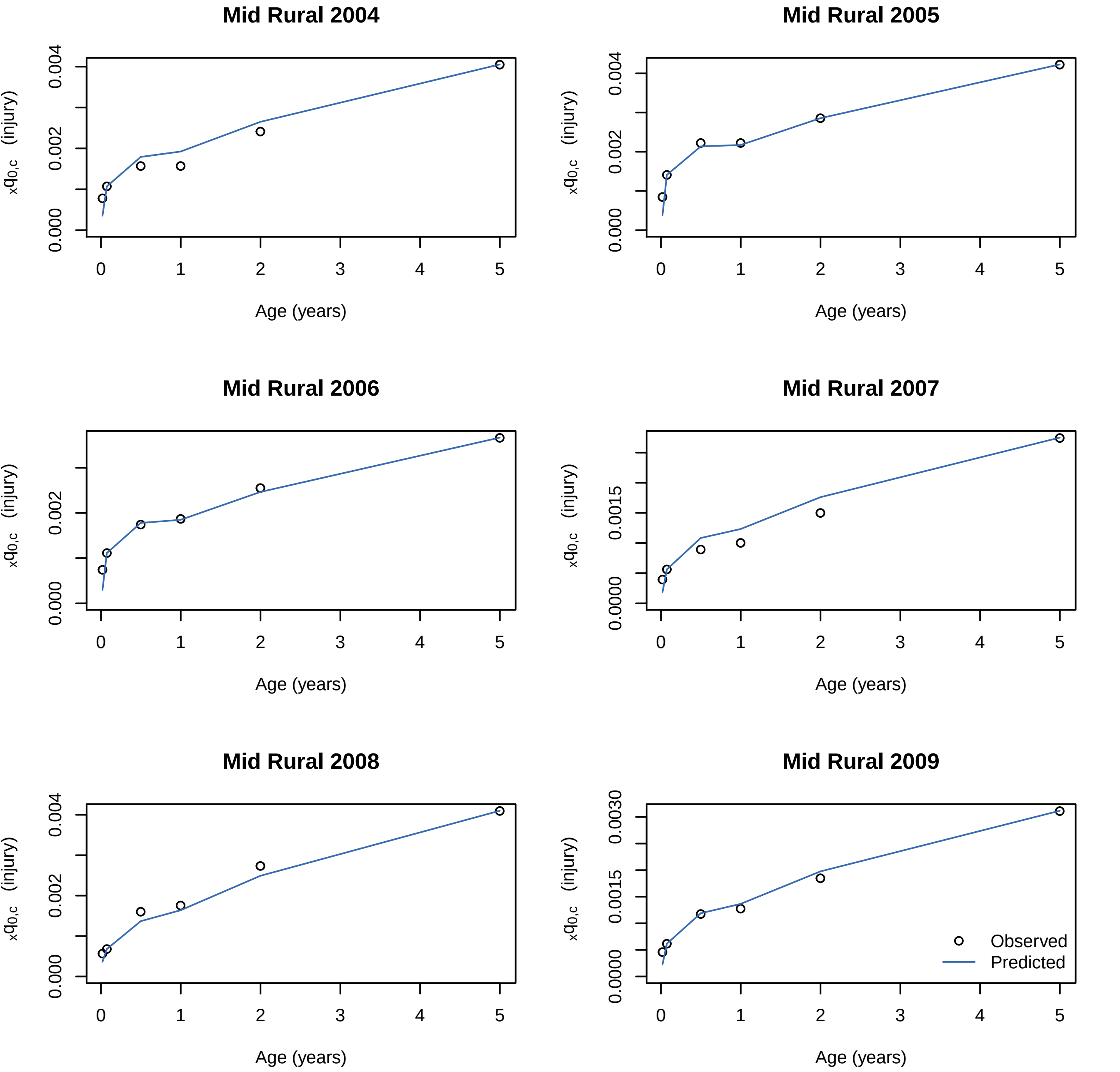

Mid Rural 2004
Mid Rural 2005
Mid Rural 2006
Mid Rural 2007
Mid Rural 2008
Mid Rural 2009
$_xq_{0,c}$ (injury)
Age (years)
0.000
0.002
0.004
0.0000
0.0015
0.0030
0
1
2
3
4
5
Observed
Predicted

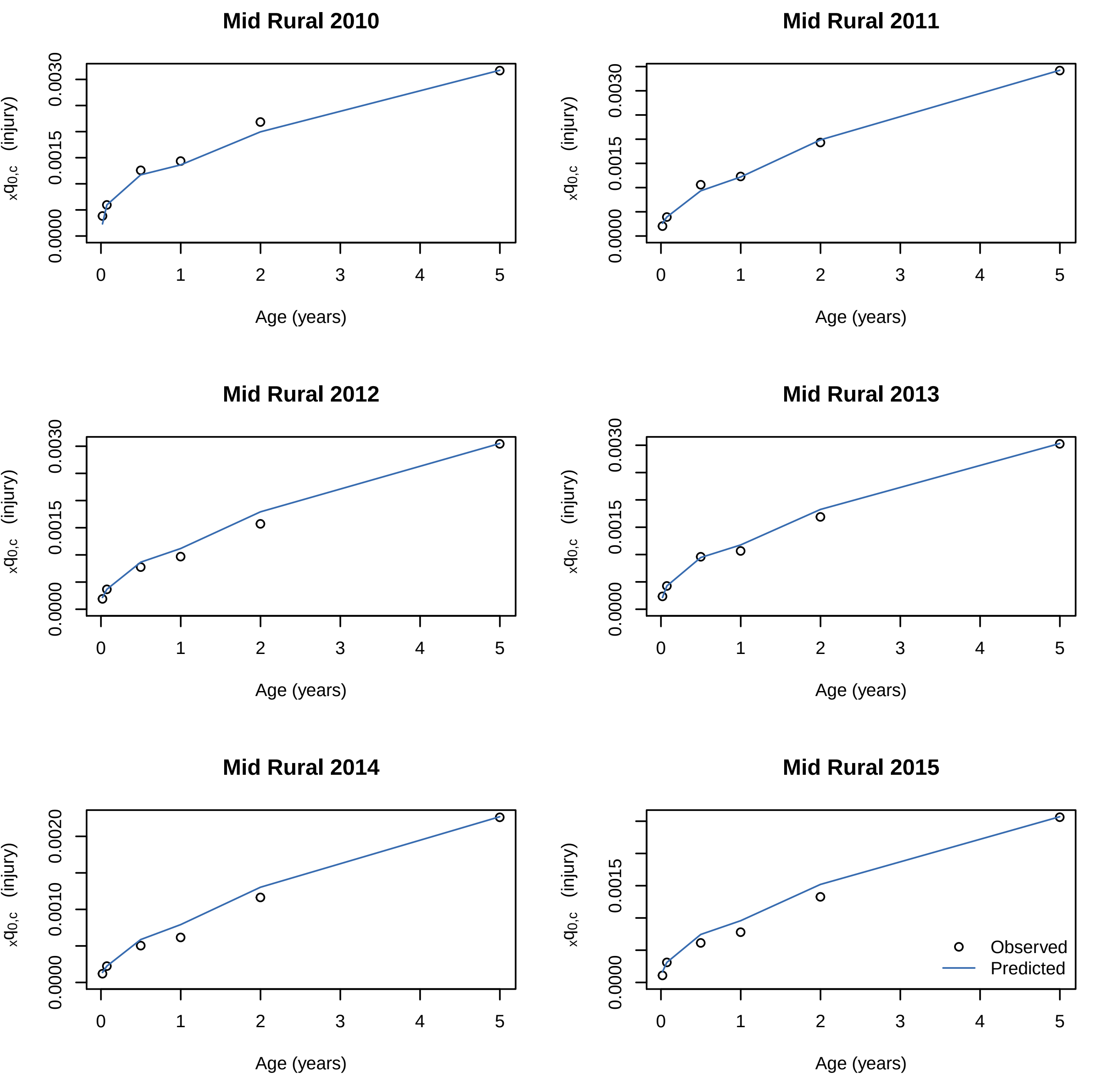

Mid Rural 2010
Mid Rural 2011
Mid Rural 2012
Mid Rural 2013
Mid Rural 2014
Mid Rural 2015
$_{x}q_{0,c}$ (injury)
Age (years)
0.0000
0.0015
0.0030
0.0010
0.0020
0 1 2 3 4 5
Observed
Predicted

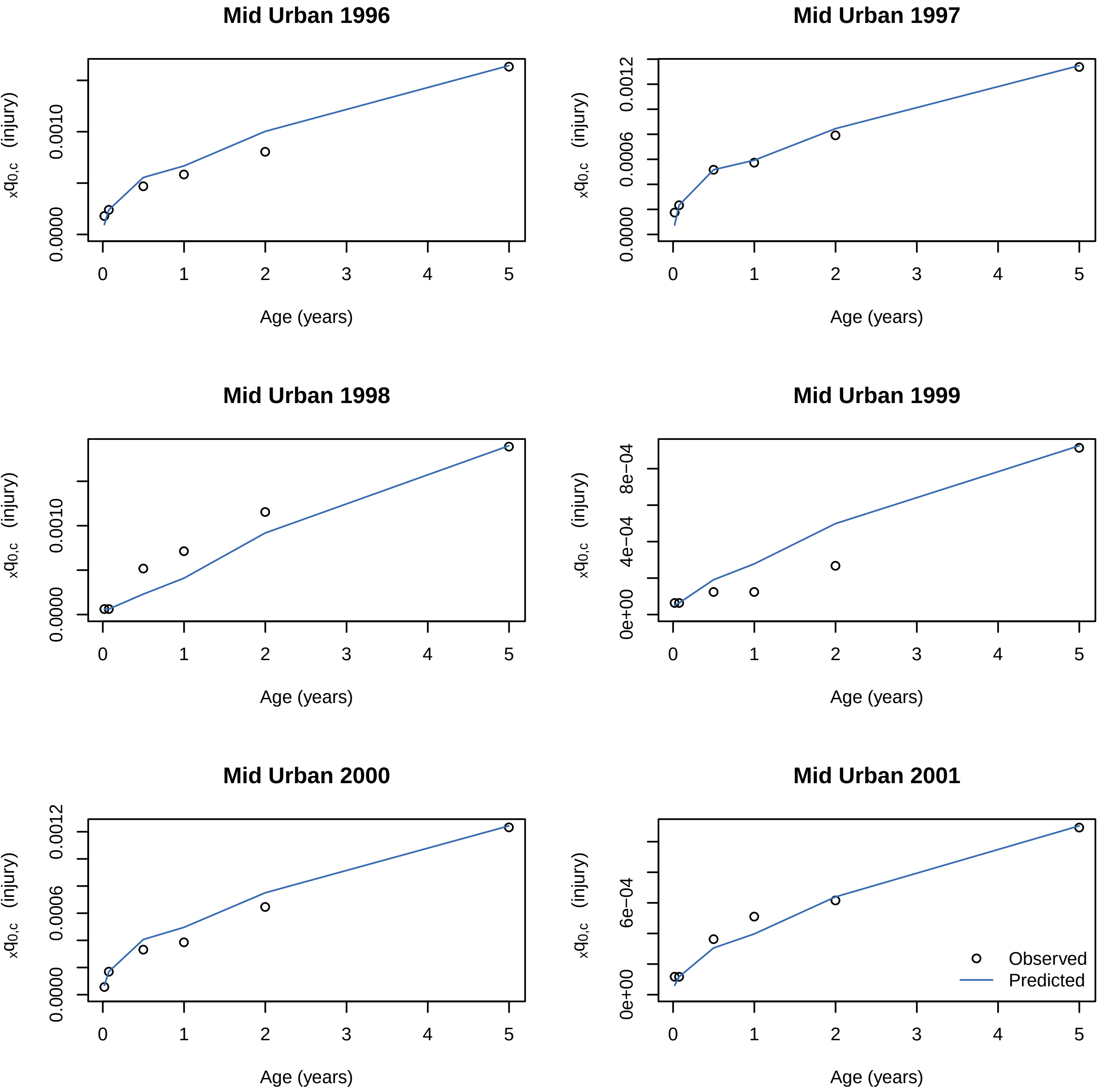

Mid Urban 1996
Mid Urban 1997
Mid Urban 1998
Mid Urban 1999
Mid Urban 2000
Mid Urban 2001
$_xq_{0,c}$ (injury)
Age (years)
0.0000
0.0010
0.0006
0.0012
0e+00
4e−04
8e−04
6e−04
0
1
2
3
4
5
Observed
Predicted

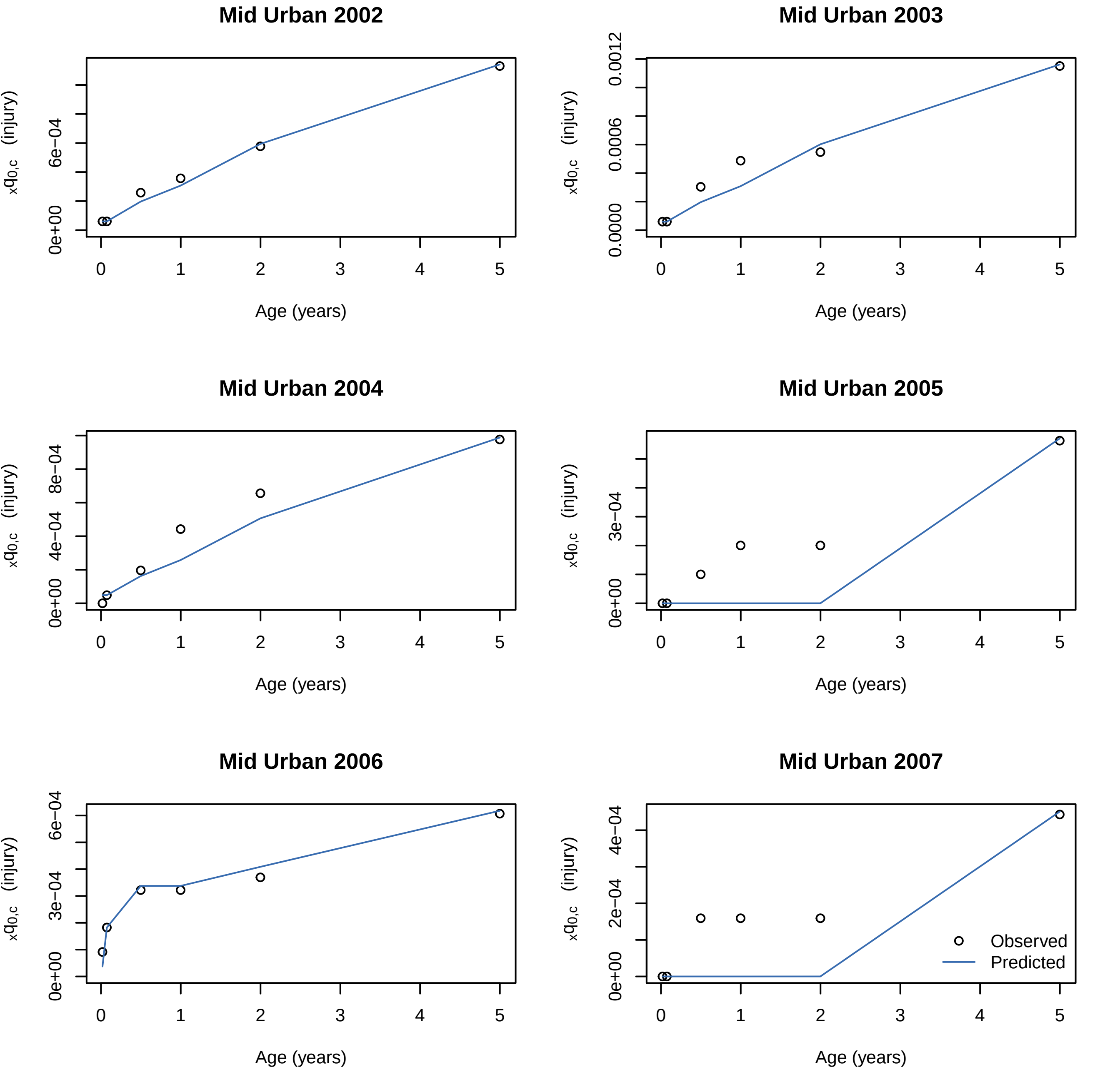

Mid Urban 2002
Mid Urban 2003
Mid Urban 2004
Mid Urban 2005
Mid Urban 2006
Mid Urban 2007
$_{x}q_{0,c}$ (injury)
Age (years)
0e+00
6e−04
0.0000
0.0006
0.0012
4e−04
8e−04
3e−04
2e−04
0
1
2
3
4
5
Observed
Predicted

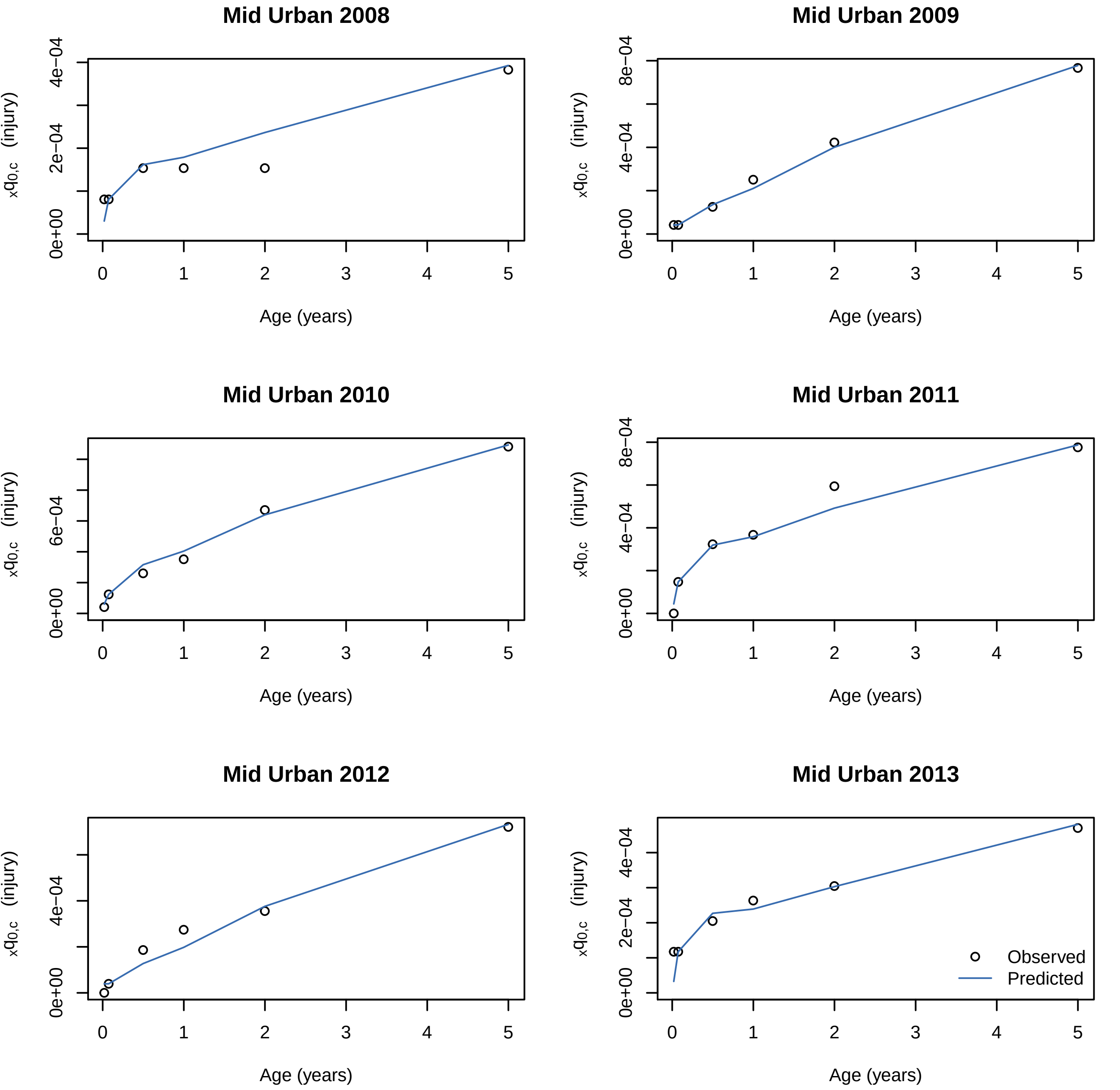

Mid Urban 2008
Mid Urban 2009
Mid Urban 2010
Mid Urban 2011
Mid Urban 2012
Mid Urban 2013
$_xq_{0,c}$ (injury)
Age (years)
0e+00
2e−04
4e−04
6e−04
8e−04
0
1
2
3
4
5
Observed
Predicted

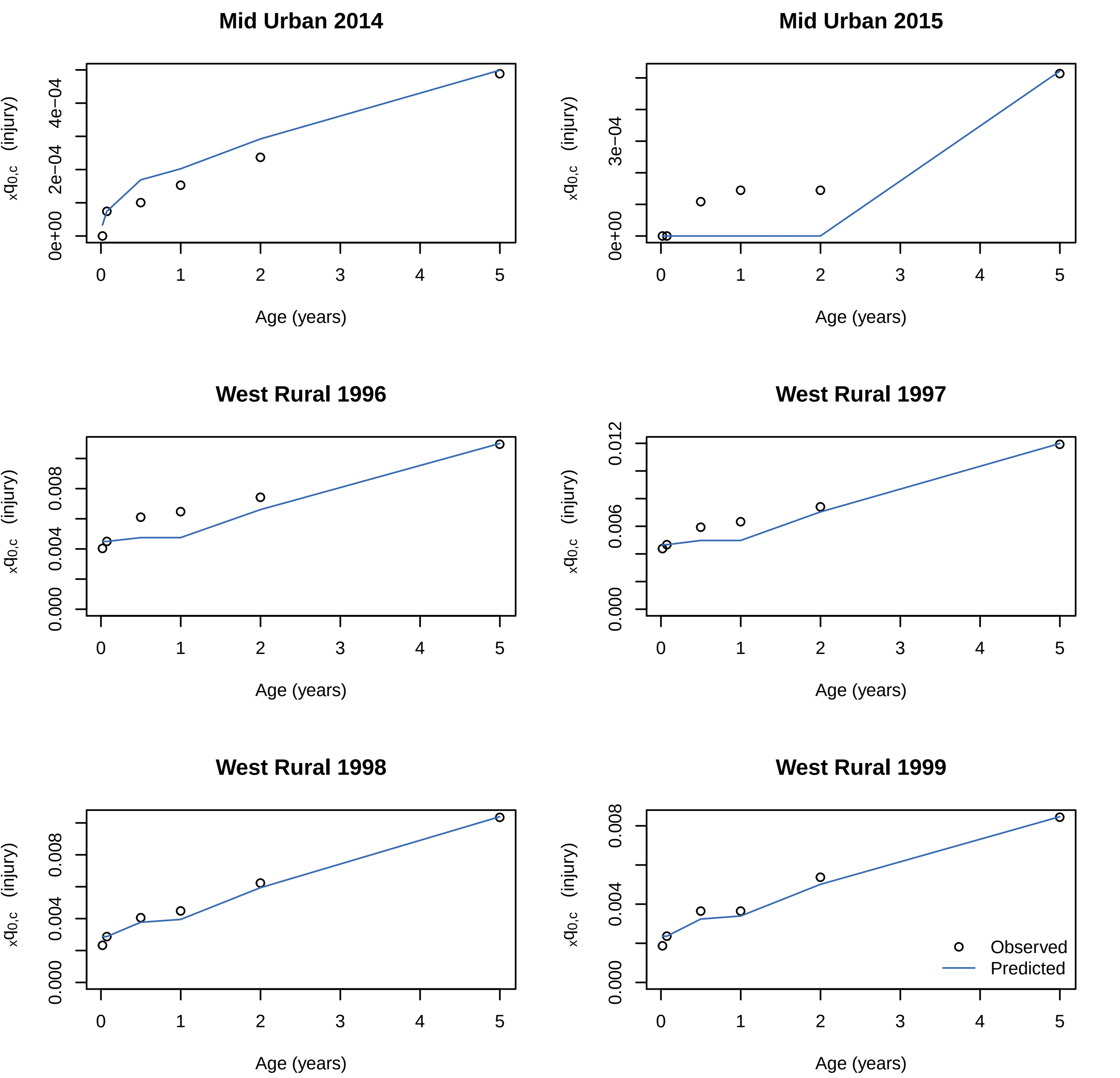

Mid Urban 2014
Mid Urban 2015
West Rural 1996
West Rural 1997
West Rural 1998
West Rural 1999
$_xq_{0,c}$ (injury)
Age (years)
0 1 2 3 4 5
0e+00 2e−04 4e−04
0e+00 3e−04
0.000 0.004 0.008
0.000 0.006 0.012
0.000 0.004 0.008
0.000 0.004 0.008
Observed
Predicted

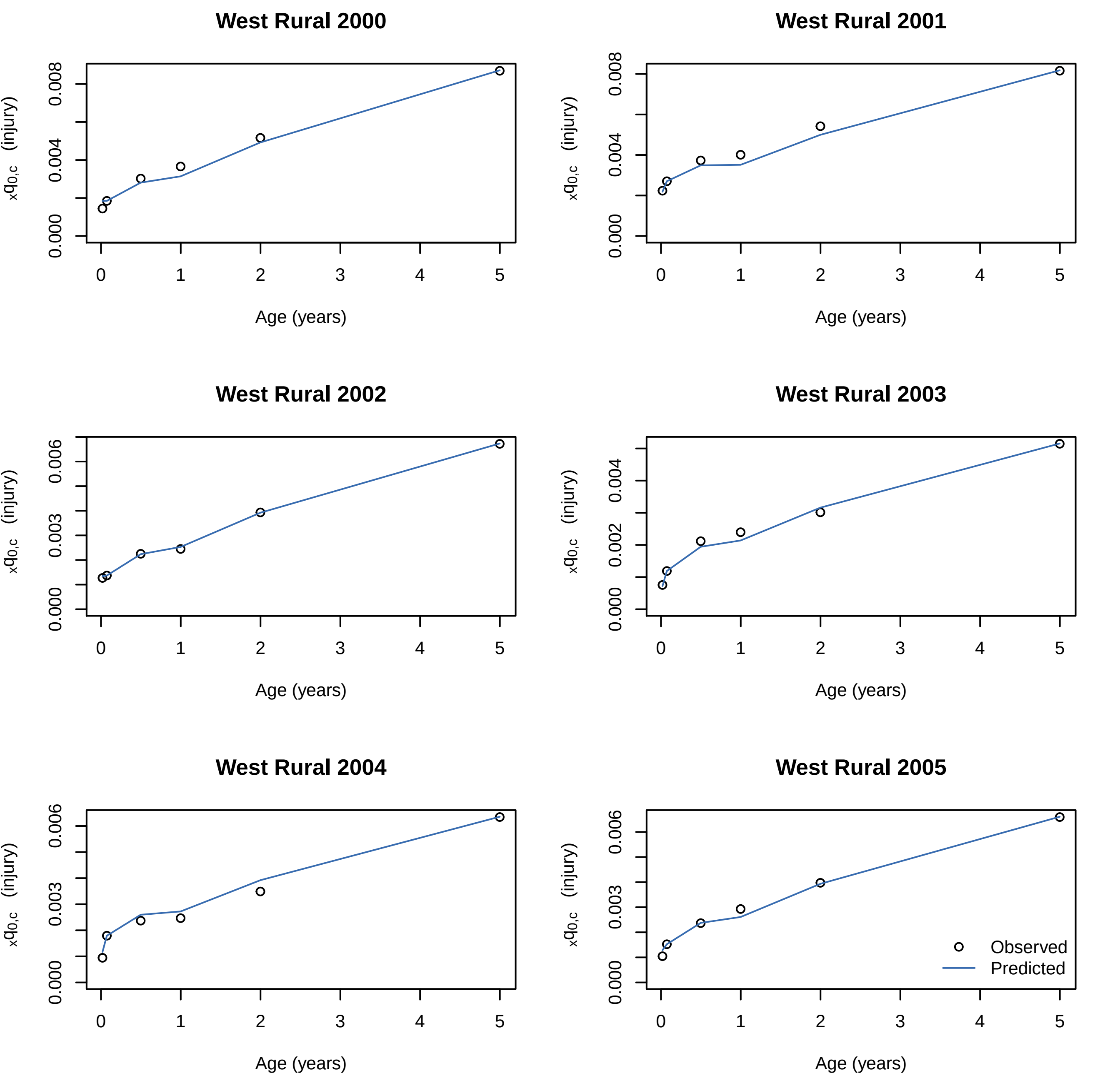

West Rural 2000
West Rural 2001
West Rural 2002
West Rural 2003
West Rural 2004
West Rural 2005
$_xq_{0,c}$ (injury)
Age (years)
0.000
0.004
0.008
0.002
0.003
0.006
0
1
2
3
4
5
Observed
Predicted

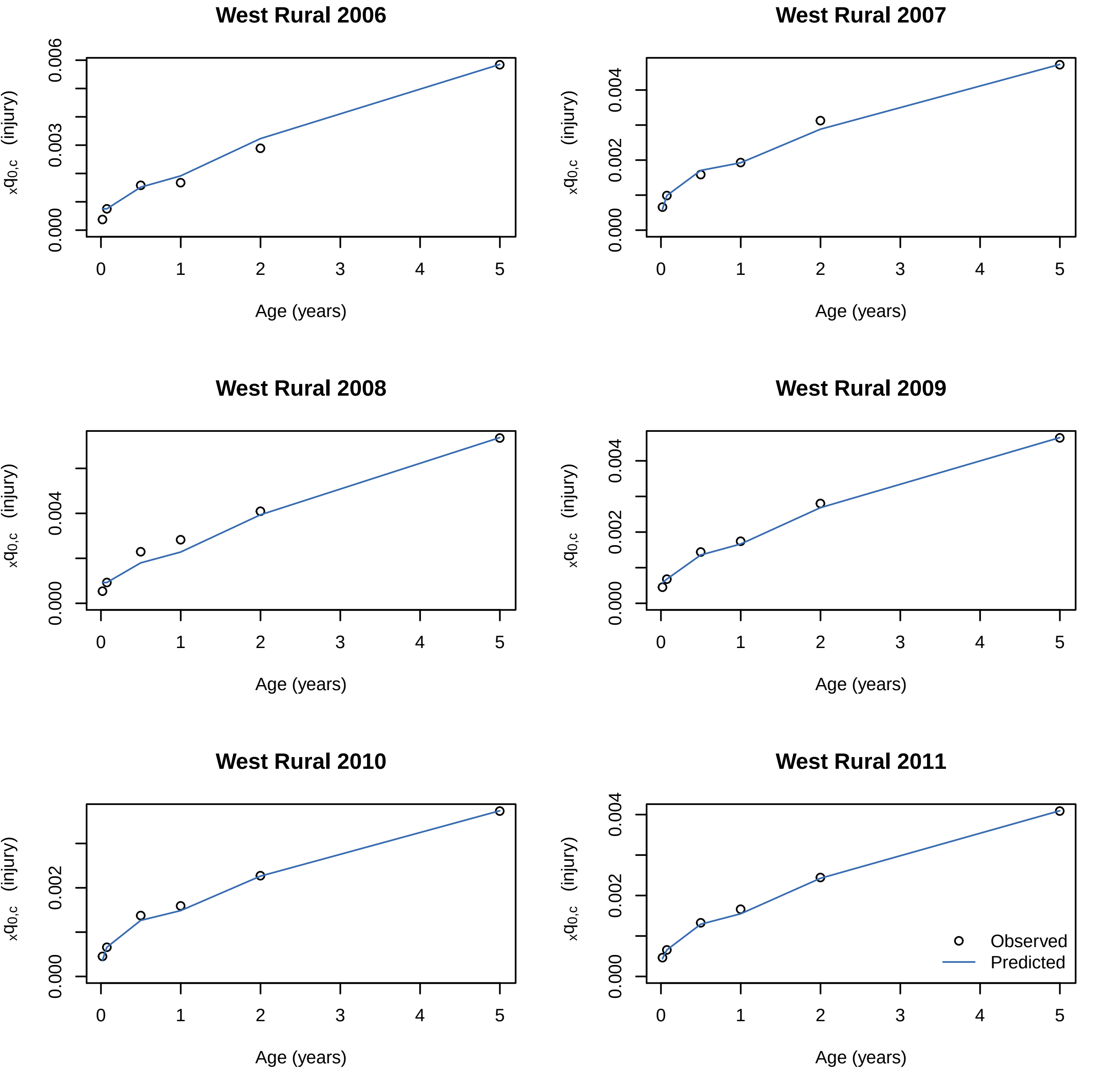
West Rural 2006
West Rural 2007
West Rural 2008
West Rural 2009
West Rural 2010
West Rural 2011
$_{x}q_{0,c}$ (injury)
Age (years)
0 1 2 3 4 5
0.000 0.003 0.006
0.000 0.002 0.004
Observed
Predicted

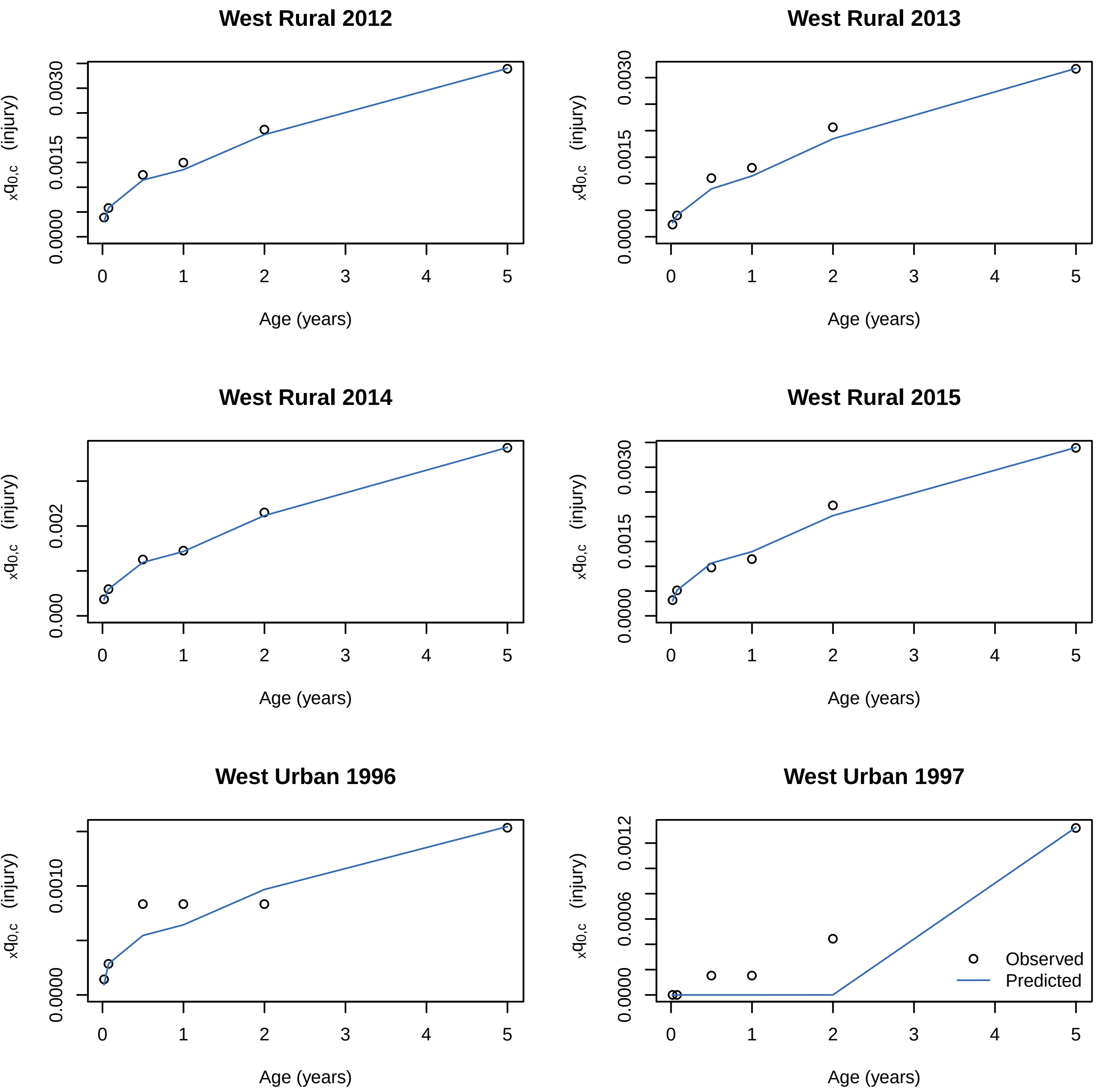
West Rural 2012
West Rural 2013
West Rural 2014
West Rural 2015
West Urban 1996
West Urban 1997
Age (years)
(injury)
0.0000
0.0015
0.0030
0.000
0.002
0.0010
0.0006
0.0012
0
1
2
3
4
5
Observed
Predicted

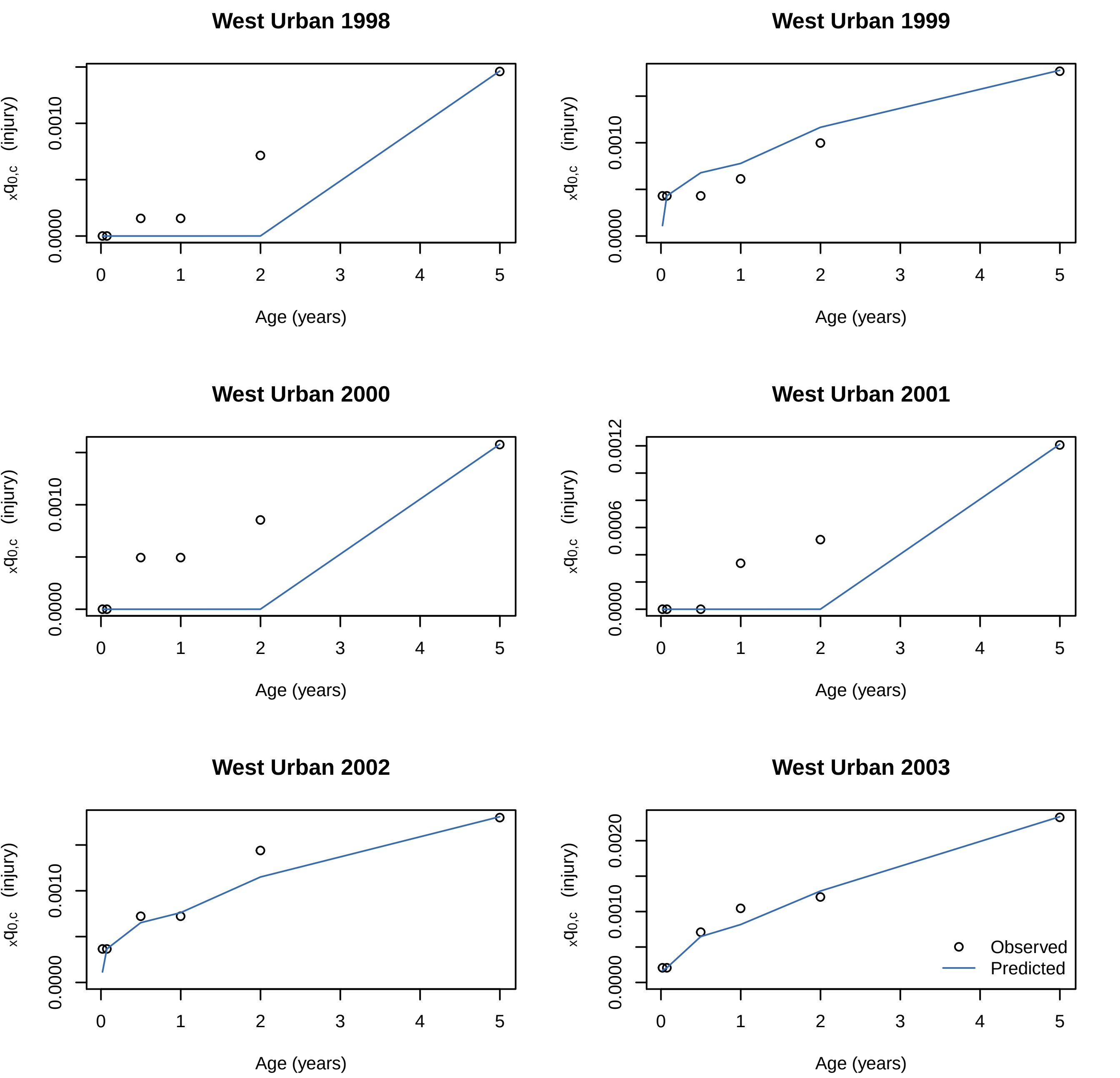
West Urban 1998
West Urban 1999
West Urban 2000
West Urban 2001
West Urban 2002
West Urban 2003
$_xq_{0,c}$ (injury)
Age (years)
0.0000
0.0010
0.0006
0.0012
0.0020
0
1
2
3
4
5
Observed
Predicted

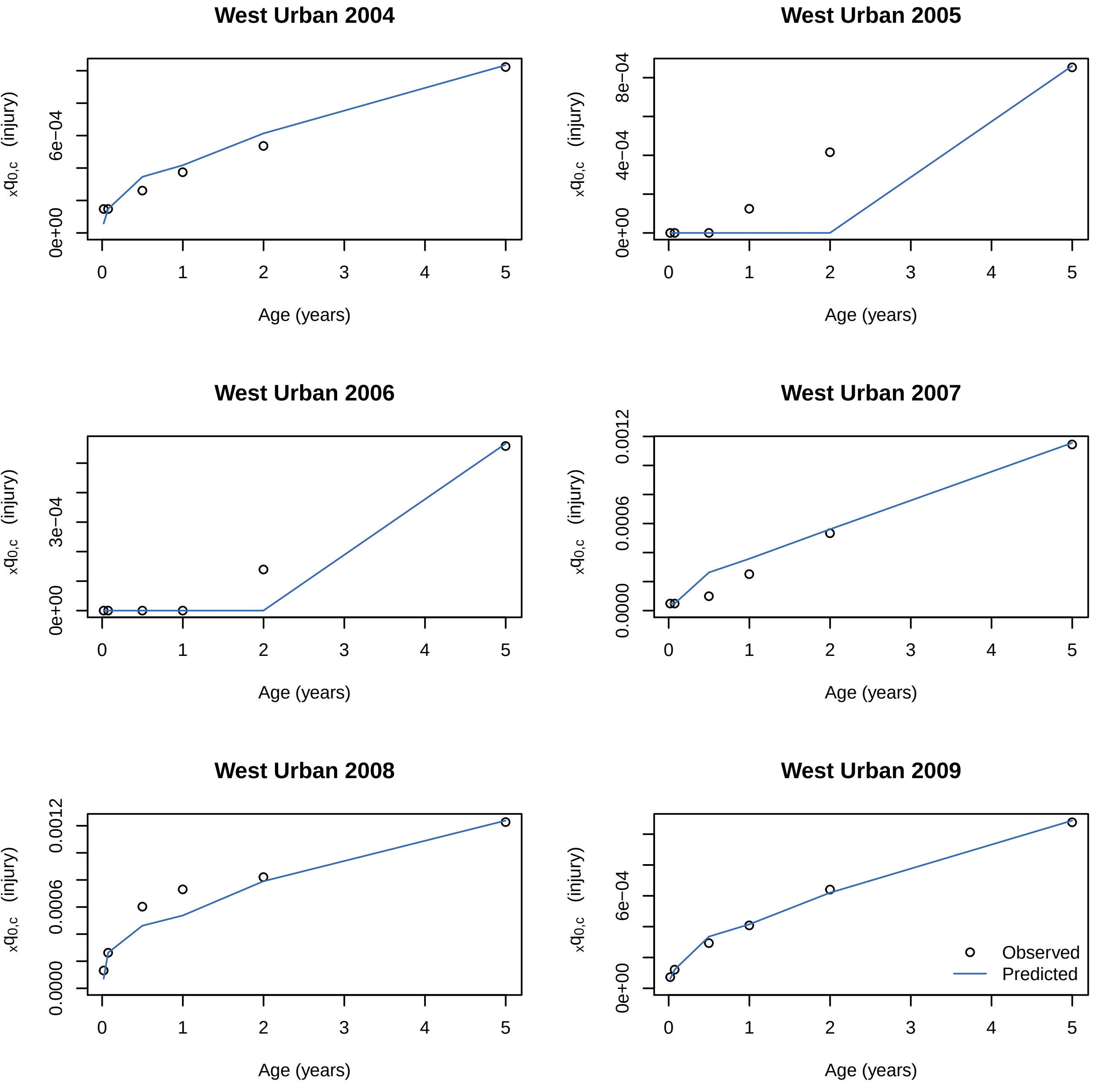
West Urban 2004
West Urban 2005
West Urban 2006
West Urban 2007
West Urban 2008
West Urban 2009
$_xq_{0,c}$ (injury)
Age (years)
0e+00
6e-04
4e-04
8e-04
3e-04
0.0000
0.0006
0.0012
0
1
2
3
4
5
Observed
Predicted

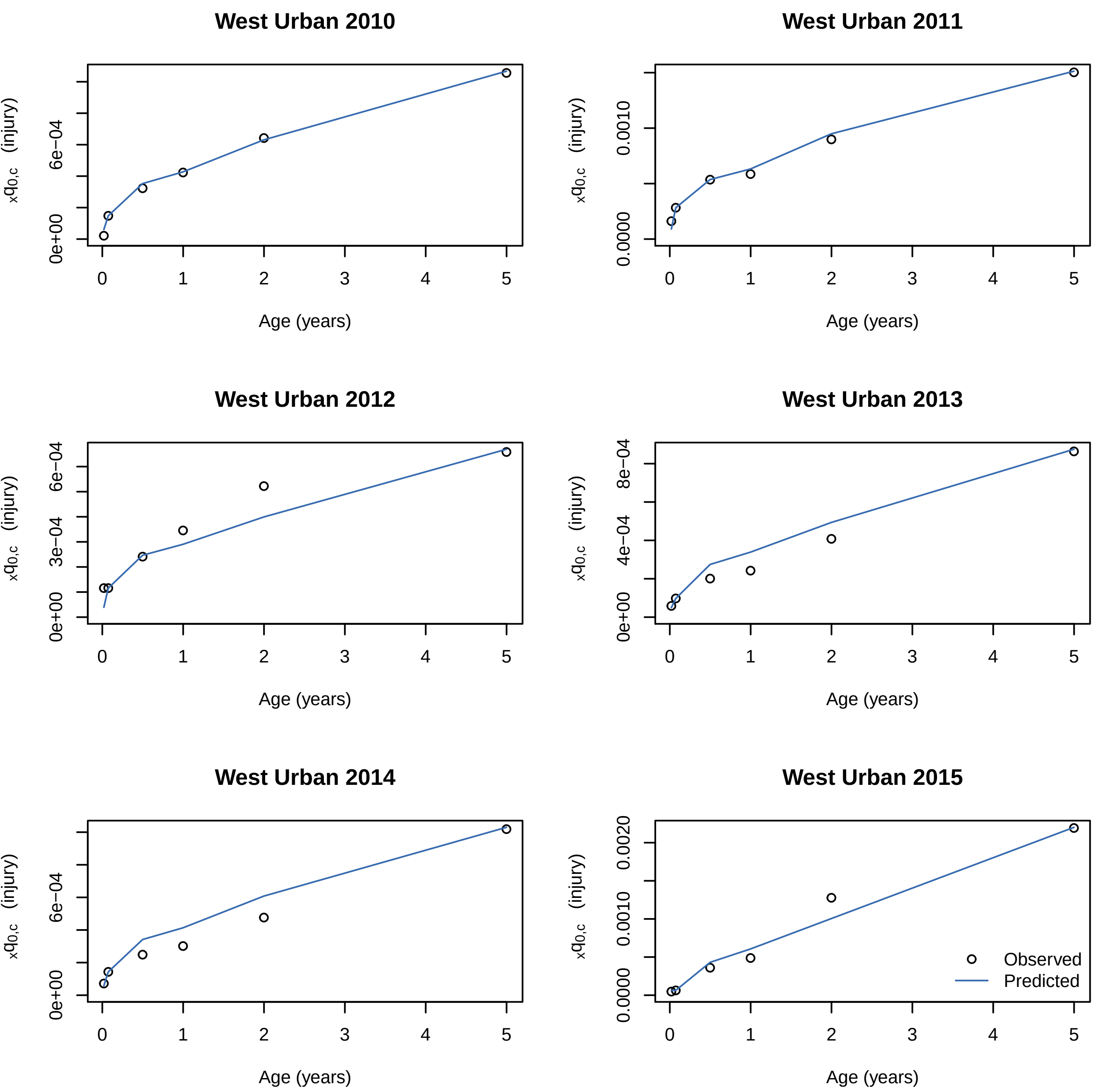

West Urban 2010
West Urban 2011
West Urban 2012
West Urban 2013
West Urban 2014
West Urban 2015
$_xq_{0,c}$ (injury)
Age (years)
0e+00
6e−04
3e−04
4e−04
8e−04
0.0000
0.0010
0.0020
0
1
2
3
4
5
Observed
Predicted
