# Supplementary material for: Adapting and validating the log quadratic model to derive under-five age- and cause-specific mortality (U5ACSM): a preliminary analysis
Source: Popul Health Metr. 2022 Jan 10;20:3. doi: 10.1186/s12963-021-00277-w (PMC8744238; doi:10.1186/s12963-021-00277-w)
Supplement: Supplementary file 4 — Additional file 4. Estimated U5ACSM using log linear model. [file 12963_2021_277_MOESM4_ESM.pdf]

Results for log-linear model

The results below are for U5ACSM estimated for 120 strata-years in China using log linear rather than the log quadratic model, as proposed. This log linear model estimates age- and cause-specific mortality using

$$\log({}_{x}q_{0,c}) = a_{x,c} + b_{x,c}\log({}_{5}q_{0,c}) + v_{x,c}k_c$$

for age x and cause c, children of ages 0–6, 7–27 days, and 1–5, 6–11, 12–23, and 24–59 months. Other conditions of this log linear model are the same as sthe log quadratic model. Please refer to the main manuscript for details.

Table 1 Relative cross validation error for single hold-out strata in all-cause, pneumonia- and injury-specific mortality by age in China, 1996-2015. Error shown as average percent difference between estimated and observed ${}_{x}q_{0}$ and ${}_{x}q_{0,c}$.

	Relative error in ${}_{x}q_{0}$ per 1000 livebirths			
Age	Log linear with k =0	Log linear with estimated k	Standard‡	${}_{x}q_{0}$ (range)
All cause†				
0-6 days (${}_{6d}q_{0}$)	21%	21%	67%	1.3 - 29.7
0-27 days (${}_{27d}q_{0}$)	16%	0%	0%	1.9 - 38.0
0-5 months (${}_{5mo}q_{0}$)	7%	4%	17%	2.6 - 55.3
0-11 months (${}_{11mo}q_{0}$)	5%	4%	18%	2.9 - 62.4
0-23 months (${}_{23mo}q_{0}$)	3%	3%	16%	3.2 - 68.4
0-59 months (${}_{59mo}q_{0}$)	0%	0%	0%	3.6 - 76.7
Pneumonia-specific*				
0-6 days (${}_{6d}q_{0,c}$)	97%	97%	51%	0.0 - 3.5
0-27 days (${}_{27d}q_{0,c}$)	45%	0%	0%	0.0 - 6.9
0-5 months (${}_{5mo}q_{0,c}$)	14%	10%	36%	0.1 - 16.0
0-11 months (${}_{11mo}q_{0,c}$)	8%	7%	37%	0.1 - 20.0
0-23 months (${}_{23mo}q_{0,c}$)	5%	5%	30%	0.2 - 22.9
0-59 months (${}_{59mo}q_{0,c}$)	0%	0%	0%	0.2 - 24.0
Injury-specific**				
0-6 days (${}_{6d}q_{0,c}$)	64%	64%	59%	0.0 - 4.4
0-27 days (${}_{27d}q_{0,c}$)	59%	0%	0%	0.0 - 4.7
0-5 months (${}_{5mo}q_{0,c}$)	39%	27%	31%	0.0 - 6.1
0-11 months (${}_{11mo}q_{0,c}$)	30%	22%	23%	0.0 - 6.5
0-23 months (${}_{23mo}q_{0,c}$)	16%	17%	18%	0.1 - 7.4
0-59 months (${}_{59mo}q_{0,c}$)	0%	0%	0%	0.3 - 11.9

‡ Based on constant mortality daily/monthly rate across age within 0-27 days and 1-59 months.

† k matched to all-cause neonatal mortality rate.

*k matched to pneumonia-specific neonatal mortality rate.

**k matched to injury-specific neonatal mortality rate.

Table 2 **Average cross validation error for single hold-out strata in all-cause, pneumonia- and injury-specific mortality by age in China, 1996-2015. Error shown as average absolute difference between estimated and observed $_{x}q_{0}$ and $_{x}q_{0,c}$.**

	Absolute error in $_{x}q_{0}$ per 1000 livebirths			
Age	Log linear with k =0	Log linear with estimated k	Standard‡	$_{x}q_{0}$ (range)
All cause†				
0-6 days ($_{6d}q_{0}$)	2.51	2.51	6.47	1.3 - 29.7
0-27 days ($_{27d}q_{0}$)	2.24	0.00	0.00	1.9 - 38.0
0-5 months ($_{5mo}q_{0}$)	1.11	0.74	2.68	2.6 - 55.3
0-11 months ($_{11mo}q_{0}$)	0.78	0.88	3.21	2.9 - 62.4
0-23 months ($_{23mo}q_{0}$)	0.44	0.62	3.00	3.2 - 68.4
0-59 months ($_{59mo}q_{0}$)	0.00	0.00	0.00	3.6 - 76.7
Pneumonia-specific*				
0-6 days ($_{6d}q_{0}$)	1.21	1.21	0.43	0.0 - 3.5
0-27 days ($_{27d}q_{0}$)	0.66	0.00	0.00	0.0 - 6.9
0-5 months ($_{5mo}q_{0}$)	0.38	0.23	1.04	0.1 - 16.0
0-11 months ($_{11mo}q_{0}$)	0.20	0.16	1.21	0.1 - 20.0
0-23 months ($_{23mo}q_{0}$)	0.11	0.10	1.08	0.2 - 22.9
0-59 months ($_{59mo}q_{0}$)	0.00	0.00	0.00	0.2 - 24.0
Injury-specific**				
0-6 days ($_{6d}q_{0}$)	0.19	0.19	0.29	0.0 - 4.4
0-27 days ($_{27d}q_{0}$)	0.22	0.00	0.00	0.0 - 4.7
0-5 months ($_{5mo}q_{0}$)	0.24	0.14	0.23	0.0 - 6.1
0-11 months ($_{11mo}q_{0}$)	0.18	0.14	0.17	0.0 - 6.5
0-23 months ($_{23mo}q_{0}$)	0.14	0.14	0.22	0.1 - 7.4
0-59 months ($_{59mo}q_{0}$)	0.00	0.00	0.00	0.3 - 11.9

‡ Based on constant mortality daily/monthly rate across age within 0-27 days and 1-59 months.

† k matched to all-cause neonatal mortality rate.

*k matched to pneumonia-specific neonatal mortality rate.

**k matched to injury-specific neonatal mortality rate.
