# Supplementary material for: Adapting and validating the log quadratic model to derive under-five age- and cause-specific mortality (U5ACSM): a preliminary analysis
Source: Popul Health Metr. 2022 Jan 10;20:3. doi: 10.1186/s12963-021-00277-w (PMC8744238; doi:10.1186/s12963-021-00277-w)
Supplement: Supplementary file 5 — Additional file 5. Sensitivity to birth cohorts in cross validation for analysis of all cause mortality. [file 12963_2021_277_MOESM5_ESM.pdf]

We describe here how death rates were estimated from the MCHSS surveillance data. We also present an alternate method for estimating death rates, and compare the proposed method for estimating age- and cause-specific mortality using both sets of estimated death rates, for all cause mortality.

Death rate estimation

We built under-five period life tables to specify observed ${}_xq_0$ and ${}_xq_{0,c}$ from recorded livebirths and deaths among children under five, using the number of live births (B) in each strata and year. The all-cause probability of mortality from birth to age x was determined by

$$ {}_xq_0 = \frac{{}_xD_0}{B} \,, $$

where ${}_xD_0$ is the total number of deaths in a given year between birth and age x. Using these age-specific probabilities of death ${}_xq_0$, age- and cause-specific probabilities were determined by

$$ {}_xq_{0,c} = {}_xq_0 \cdot \frac{{}_xD_{0,c}}{{}_xD_0} \,, $$

where ${}_xD_{0,c}$ represents the number of deaths for cause c between birth and age x.

Alternate death rate estimation with birth cohort reconstruction

First, we calculate the number of individuals alive in each age group at the end of each time interval ($N_x(t)$ values of the Lexis diagram) by reconstructing birth cohorts from survey data. The procedure works as follows:

1 Six age groups are available in the MCHSS for children under five: 0-6 days, 7-27 days, 1-5 months, 6-11 months, 12-23 months, and 24-59 months.

2 Because data is retrieved in annual basis, some adjustments are necessary. Assuming the exposures to be proportional to the length of the age interval, we calculated the following "proportions of deaths within each age interval that correspond to individuals who entered the age interval that calendar year": $p_{(0-6d)} = 0.990418$; $p_{(7-27d)} = 0.952088$; $p_{(1-5m)} = 0.71167$; and $p_{(6-11m)} = 0.25$.

3 For a given year t, using these proportions together with the number of births $B(t)$ and deaths below age 1 from that year, we obtain the number individuals in age 0 to 11 months at the end of calendar year t,

$$ \begin{aligned} N_{(0-11m)}(t) \quad = \quad & B(t) - D_{(0-6d)}(t) \times p_{(0-6d)} - D_{(7-27d)}(t) \times p_{(7-27d)} - \\ & D_{(1-5m)}(t) \times p_{(1-5m)} - D_{(6-11m)}(t) \times p_{(6-11m)}. \end{aligned} $$

4 That way, we obtain $N_{(0-11m)}(t)$ estimates for the end of years 1996 to 2015.

5 Assuming exposures to be proportional to the length of the age intervals, we calculate the $N_x(t)$ values for finer age groups: 0-6 days, 7-27 days, 1-5 months, and 6-11 months.

6 Next, using the $N_{(0-11m)}(t)$ estimates and the corresponding deaths in the following calendar year t+1, we calculate the number of individuals in age 12 to 23 months at the end of $t+1$, given by

$$\begin{aligned} N_{(12-23m)}(t+1) \quad = \quad & N_{(0-11m)}(t) - D_{(0-6d)}(t+1) \times (1 - p_{(0-6d)}) - \\ & D_{(7-27d)}(t+1) \times (1 - p_{(7-27d)}) - D_{(1-5m)}(t+1) \times (1 - p_{(1-5m)}) - \\ & D_{(6-11m)}(t) \times (1 - p_{(6-11m)}) - D_{(12-23m)}(t) \times 0.5. \end{aligned}$$

7 That way, we obtain $N_{(12-23m)}(t)$ estimates for the end of years 1997 to 2015.

8 Finally, following a similar procedure and assuming deaths are distributed equally within each calendar year, we calculate $N_{(24-59m)}(t)$, the number of individuals alive in ages 24 to 59 months a the end of calendar years 2000 to 2015.

As a result, for years 2000 to 2015 we get $N_x(t)$ estimates for all six age groups: 0-6 days, 7-27 days, 1-5 months, and 6-11 months, 12-23 months, and 24-59 months. However, because of the surveillance expansion, data from 2008 or earlier cannot be combined with data from 2009 onwards. Accordingly, we dropped estimates from 2009-2012.

In a second step, we calculate the mid-year population for each age group x, given by

$$L_x(t) = \frac{N_x(t-1) + N_x(t)}{2},$$

for t=2001-2008, 2014, 2015. Finally, for these 10 years and 6 age groups we get the age-specific central death rates

$$M_x(t) = \frac{D_x(t)}{L_x(t)}.$$

Additional details, as well as the R code, are available upon request.

Results

Cross validation results are shown below for two sets of all cause death rates among children under five by age group. While cross validation error is somewhat reduced when estimating mortality using the alternate method, the proposed method has lower cross validation error for both sets of estimated death rates, by several orders of magnitutde.

Table 1 Relative cross validation error for single hold-out strata in all-cause, pneumonia- and injury-specific mortality by age in China, 1996-2015. Error shown as average percent difference between estimated and observed $_{x}q_{0}$ and $_{x}q_{0,c}$.

	Relative error in $_{x}q_{0}$ per 1000 livebirths			
Age	Log linear with k =0	Log linear with estimated k	Standard[‡]	$_{x}q_{0}$ (range)
All cause (1996-2015)[†]				
0-6 days ($_{6d}q_{0}$)	21%	21%	67%	1.3 - 29.7
0-27 days ($_{27d}q_{0}$)	16%	0%	0%	1.9 - 38.0
0-5 months ($_{5mo}q_{0}$)	7%	4%	17%	2.6 - 55.3
0-11 months ($_{11mo}q_{0}$)	5%	4%	18%	2.9 - 62.4
0-23 months ($_{23mo}q_{0}$)	3%	3%	16%	3.2 - 68.4
0-59 months ($_{59mo}q_{0}$)	0%	0%	0%	3.6 - 76.7
All cause (estimated with birth cohort reconstruction, 2001-2008 & 2014-2015)[†]				
0-6 days ($_{6d}q_{0}$)	18%	18%	67%	0.0 - 3.5
0-27 days ($_{27d}q_{0}$)	14%	0%	0%	0.0 - 6.9
0-5 months ($_{5mo}q_{0}$)	8%	3%	16%	0.1 - 16.0
0-11 months ($_{11mo}q_{0}$)	6%	4%	17%	0.1 - 20.0
0-23 months ($_{23mo}q_{0}$)	4%	3%	15%	0.2 - 22.9
0-59 months ($_{59mo}q_{0}$)	0%	0%	0%	0.2 - 24.0

‡ Based on constant mortality daily/monthly rate across age within 0-27 days and 1-59 months.

† k matched to all-cause neonatal mortality rate.

Table 2 Average cross validation error for single hold-out strata in all-cause, pneumonia- and injury-specific mortality by age in China, 1996-2015. Error shown as average absolute difference between estimated and observed $_{x}q_{0}$ and $_{x}q_{0,c}$.

	Absolute error in $_{x}q_{0}$ per 1000 livebirths			
Age	Log linear with k =0	Log linear with estimated k	Standard[‡]	$_{x}q_{0}$ (range)
All cause (1996 - 2015)[†]				
0-6 days ($_{6d}q_{0}$)	2.51	2.51	6.47	1.3 - 29.7
0-27 days ($_{27d}q_{0}$)	2.24	0.00	0.00	1.9 - 38.0
0-5 months ($_{5mo}q_{0}$)	1.11	0.74	2.68	2.6 - 55.3
0-11 months ($_{11mo}q_{0}$)	0.78	0.88	3.21	2.9 - 62.4
0-23 months ($_{23mo}q_{0}$)	0.44	0.62	3.00	3.2 - 68.4
0-59 months ($_{59mo}q_{0}$)	0.00	0.00	0.00	3.6 - 76.7
All cause (estimated by birth cohort reconstruction, 2001-2008 & 2014-2015)[†]				
0-6 days ($_{6d}q_{0}$)	1.87	1.87	6.21	0.0 - 3.5
0-27 days ($_{27d}q_{0}$)	1.87	0.00	0.00	0.0 - 6.9
0-5 months ($_{5mo}q_{0}$)	1.11	0.61	2.46	0.1 - 16.0
0-11 months ($_{11mo}q_{0}$)	0.83	0.74	2.87	0.1 - 20.0
0-23 months ($_{23mo}q_{0}$)	0.50	0.58	2.61	0.2 - 22.9
0-59 months ($_{59mo}q_{0}$)	0.00	0.00	0.00	0.2 - 24.0

‡ Based on constant mortality daily/monthly rate across age within 0-27 days and 1-59 months.

† k matched to all-cause neonatal mortality rate.
